# Mitochondrial Genomes Provide Insights into the Phylogeny of Lauxanioidea (Diptera: Cyclorrhapha)

**DOI:** 10.3390/ijms18040773

**Published:** 2017-04-14

**Authors:** Xuankun Li, Wenliang Li, Shuangmei Ding, Stephen L. Cameron, Meng Mao, Li Shi, Ding Yang

**Affiliations:** 1 Department of Entomology, China Agricultural University, Beijing 100193, China; xuankun.li@csiro.au (X.L.); shuangmeiding@cau.edu.an (S.D.); 2 College of Forestry, Henan University of Science and Technology, Luoyang 471023, China; wenliangli@haust.edu.cn; 3 Department of Entomology, Purdue University, West Lafayette, IN 47907, USA; cameros@purdue.edu; 4 Department of Plant and Environmental Protection Science, University of Hawaii at Manoa, Honolulu, HI 96822, USA; mm663@uowmail.edu.au; 5 College of Agronomy, Inner Mongolia Agricultural University, Hohhot 010018, China

**Keywords:** Lauxanioidea, Cyclorrhapha, mitochondrial genome, phylogeny, RNAs, intergenic sequences

## Abstract

The superfamily Lauxanioidea is a significant dipteran clade including over 2500 known species in three families: Lauxaniidae, Celyphidae and Chamaemyiidae. We sequenced the first five (three complete and two partial) lauxanioid mitochondrial (mt) genomes, and used them to reconstruct the phylogeny of this group. The lauxanioid mt genomes are typical of the Diptera, containing all 37 genes usually present in bilaterian animals. A total of three conserved intergenic sequences have been reported across the Cyclorrhapha. The inferred secondary structure of 22 tRNAs suggested five substitution patterns among the Cyclorrhapha. The control region in the Lauxanioidea has apparently evolved very fast, but four conserved structural elements were detected in all three complete mt genome sequences. Phylogenetic relationships based on the mt genome data were inferred by Maximum Likelihood and Bayesian methods. The traditional relationships between families within the Lauxanioidea, (Chamaemyiidae + (Lauxaniidae + Celyphidae)), were corroborated; however, the higher-level relationships between cyclorrhaphan superfamilies are mostly poorly supported.

## 1. Introduction

The mitochondrion (mt), one of the fundamental eukaryotic organelles, is descended from an α-proteobacterium and as such retains a remnant, bacterial-like genome [1,2,3]. The mt genome has been widely used as an estimator for phylogentic studies, mainly because: (1) the high copy number and commonly available conserved primer sets make them easy to obtain [4]; and (2) they have enough phylogenetic information for inference over extensive taxonomic scales (e.g., [5,6,7,8,9]). Since the first insect mt genome was published by Clary and Wolstenholme in 1985 [10], the number of sequenced insect mt genomes has risen rapidly and mt genomes are available from every insect order [2]. The Diptera (flies) are one of the most extensively sequenced orders amongst the Insecta, with 115 complete, nearly-complete or partial mt genomes in GenBank (as of 1 July 2015) (Table 1). Note, here we define nearly-complete genomes as those for which none or only part of the control region has been sequenced; partial genomes are those with all 13 PCGs (protein-encoding genes) sequenced but for which one or more tRNA or rRNA genes remain unsequenced. Mt genomes with none of the 13 PCGs completely sequenced were excluded from the above statistics and the following comparative analyses.

The dipteran superfamily Lauxanioidea was first proposed by Hendel [40,41] and includes three families: Lauxaniidae, Celyphidae and Chamaemyiidae. Two additional families, Periscelididae and Eurychoromyiidae, were added into this group by Hennig [42], but he later excluded the Periscelididae [43] and placed the Eurychoromyiidae into the Sciomyzoidea [44]. The Eurychoromyiidae was recently combined into the Lauxaniidae [45], and therefore the superfamily is still composed of the three original families proposed by Hendel [40,41]. The Lauxaniidae, including 172 genera and 2150 known species, is one of the most diverse families of acalyptrate flies, and occurs on all continents except for Antarctica. As they are sensitive to pesticides and fungicides, lauxaniid flies have been used to evaluate environmental change in field ecosystems [46]. The Celyphidae, commonly known as beetle flies, are one of the most easily recognized fly families with a shiny, enlarged, elytra-like scutellum that covers most of the abdomen. It is a relatively small family with about 120 known species, and mostly occurs in the Oriental bioregion. Larvae of most Lauxaniidae and Celyphidae species have similar habits, feeding on decaying leaves or grass, while some Lauxaniidae species occur only in birds’ nests [47]. The Chamaemyiidae, on the other hand, have a very different habit from the above two families—all known larvae feed on aphids and scale insects. There are around 350 known species of Chamaemyiidae worldwide, but they are infrequently collected [47]. Because of their importance as predators of aphids, they are also called “aphid files.” Some species are even used as natural enemies in biological control measures. Currently, no mt genomes have been reported from any members of this superfamily.

The major synapomorphies for the group are convergent post-ocellar bristles, an abbreviated anal vein, and that the male abdominal tergites 7 and 8 are fused [48]. All early major classifications [41,43,49,50] as well as the recent major phylogenetic synthesis of flies [51] supported the monophyly of the Lauxanioidea. However, the phylogeny of this group has been the subject of a long-lasting, contentious debate. Hennig [52] proposed that the sister group of the Lauxanioidea was probably the Sciomyzoidea. Griffiths [50], however, concluded that the Lauxanioidea and Sciomyzoidea were only remotely related, and suggested that the Schizophora include the Lonchaeoidea, Lauxanioidea, Drosophiloidea and Nothyboidea. McAlpine [49] resolved the phylogenetic arrangement of the Acalyptratae, which supported Hennig [52] in finding that the Lauxanioidea and Sciomyzoidea were sister groups. This solution was also supported by the supertree analysis of Yeates et al. [48]. Weigmann et al. [51] suggested a sister relationship between the clades (Tephritidae + Sepsidae) and (Diopsidae + Lauxaniidae). Conversely, morphological data weakly supported the clade (Lauxaniidae + (Agromyzidae + (Chloropidae + (Drosophilidae + Sphaeroceridae)))) [53]. The aim of this current study was to test the monophyly and intraordinal placement of the Lauxanioidea using mitochondrial genome data, a data source that has proven valuable in resolving relationships between fly families in many previous studies (e.g., [30,31]).

## 2. Results and Discussion

### 2.1. General Features of Mitochondrial Genome Organization

**Figure 1:**
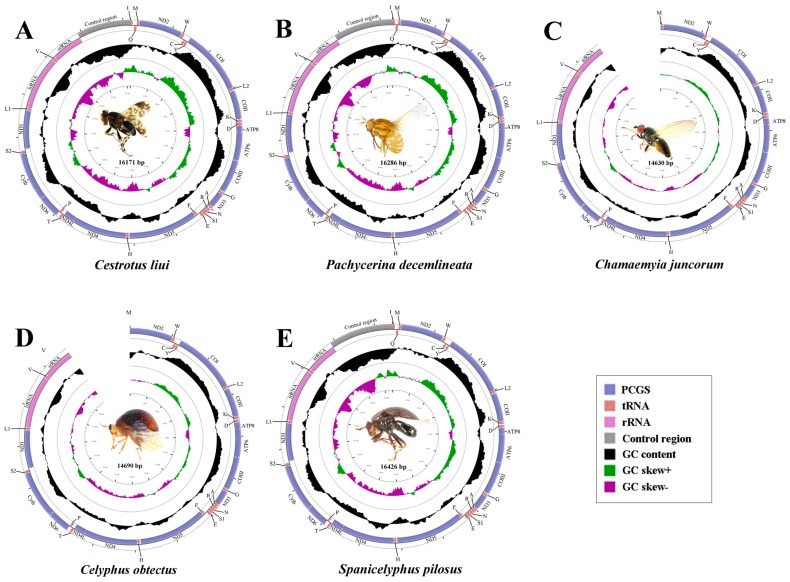
Mitochondrial genomes of five lauxaniid flies sequenced in this study. (**A**) *Cestrotus liui*; (**B**) *Pachycerina decemlineata*; (**C**) *Chamaemyia juncorum*; (**D**) *Celyphus obtectus*; (**E**) *Spanicelyphus pilosus*. Circular maps were drawn with CGView [55]. Arrows indicated the orientation of gene transcription. The tRNAs are denoted by the color blocks and are labelled according to the IUPAC-IUB single-letter amino acid codes (L1: CUN; L2: UUR; S1: AGN; S2: UCN). The guanine-cytosine (GC) content was plotted using a black sliding window, as the deviation from the average GC content of the entire sequence. GC-skew was plotted as the deviation from the average GC-skew of the entire sequence. The inner cycle indicated the location of genes in the mt genome.

**Figure 2:**
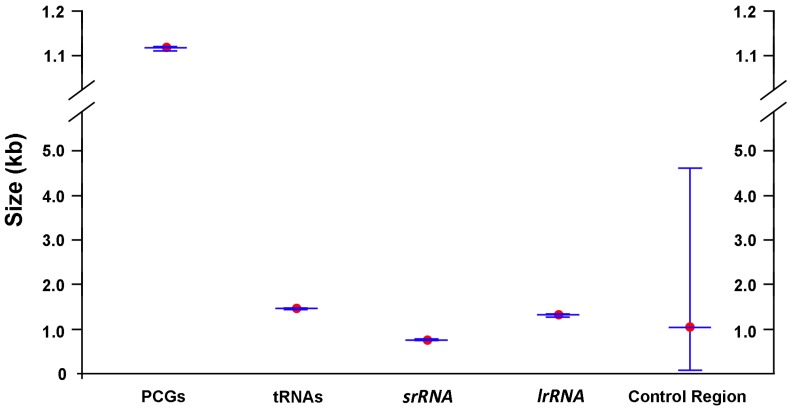
The size of protein-encoding genes (PCGs), tRNAs, *srRNA*, *lrRNA* and control region, respectively, among the sequenced Cyclorrhapha mt genomes.

In this study, the mt genomes of five lauxanioid flies, including two complete mt genomes of the Lauxaniidae, one complete and one partial mt genome of the Celyphidae and one partial mt genome of the Chamaemyidae, were sequenced for the first time (Figure 1). The sequenced mt genomes are typically circular, double-stranded molecules, containing the 37 genes (13 PCGs, 22 tRNA genes, and two rRNA genes) and a large control region (in arthropods, also known as A + T-rich region), which are usually present in bilaterian animals [2]. The length of the three complete mt genomes are 16,171 bp in *Cestrotus liui*, 16,286 bp in *Pachycerina decemlineata* and 16,426 bp in *Spanicelyphus pilosus*. They are medium-sized when compared with the mt genomes of other Cyclorrhapha, which range from 14,903 bp (*Aldrichina grahami*, Calliphoridae) [54] to 19,517 bp (*Drosophila melanogaster*, Drosophilidae) [26]. Within cyclorrhaphan mt genomes, length variation is limited in the PCGs, tRNA, and rRNA genes, but there is remarkable variation in the size of the control region (Figure 2). Mitochondrial gene pattern is the same as all previously published cyclorrhaphan mt genomes, as well as that of the inferred ancestral insect mt genome order. Majority strand (J-strand) includes 23 genes, while the remaining 14 genes are located on the minority strand (N-strand) encodes. 

### 2.2. Base Composition

**Figure 3:**
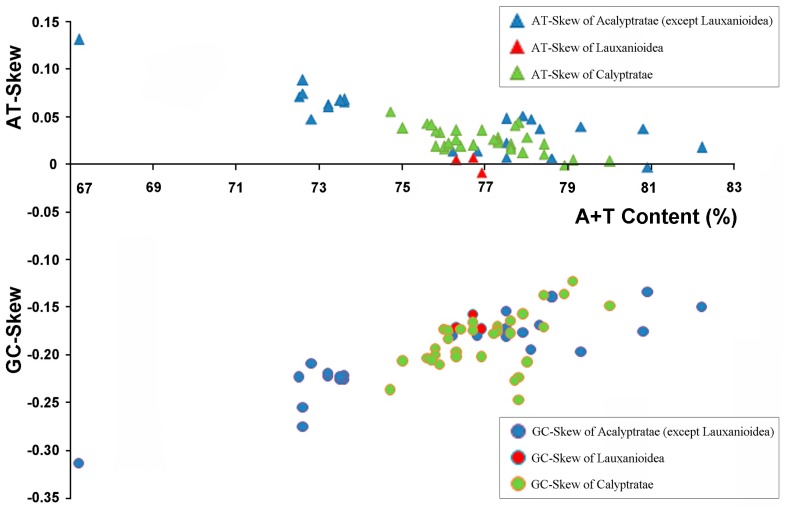
AT% vs. AT-Skew and GC% vs. AT-Skew in Cyclorrhapha mt genomes. Measured in bp percentage (*X*-axis) and level of nucleotide skew (*Y*-axis). Values are calculated on full length mt genomes. Blue, Acalyptratae (except Lauxanioidea); Red, Lauxanioidea; Green, Calyptratae; triangle, AT% vs. AT-Skew; circle, GC% vs. AT-Skew.

The nucleotide composition of the three complete lauxanioid sequences was biased toward A and T, with the overall A + T content of the mt genomes ranging from 76.3% (in *Pachycerina decemlineata*, Lauxaniidae, present study) to 76.9% (in *Spanicelyphus pilosus*, Celyphidae, present study), with an intermediate value with respect to all reported cyclorrhaphan flies, which range from 67.2% (in *Bactrocera minax*, Tephritidae [20]) to 82.2% (in *Drosophila melanogaster*, Drosophilidae [26]). Most sequenced mt genomes of other cyclorrhaphan flies present a positive AT-skew for the J-strand with an average of 0.032 (except for in *Stomoxys calcitrans* (−0.001) [31] and in *Simosyrphus grandicornis* (−0.004) [12]), ranging from −0.004 (*Simosyrphus grandicornis*, Syrphidae [12]) to 0.131 (*Bactrocera minax*, Tephritidae [20]), whereas the AT-skew of the lauxanioid mt genomes were relatively low, ranging from −0.009 (*Spanicelyphus pilosus*, Celyphidae, present study) to 0.007 (*Cestrotus liui*, Lauxaniidae, present study). The average GC-skew of other cyclorrhaphan mt genomes was −0.190, ranging from −0.315 (*Bactrocera minax*, Tephritidae [20]) to −0.124 (*Haematobia irritans*, Muscidae [31]), while the GC-skew of the lauxanioid mt genomes—which ranges from −0.159 (*Cestrotus liui*, Lauxaniidae, present study) to −0.174 (*Spanicelyphus pilosus*, Celyphidae, present study)—were average amongst reported cyclorrhaphan flies (Figure 3). In most metazoan mt genomes, the strand skew biases are found to be weakly positive AT-skew and strongly negative GC-skew for the J-strand. This pattern is consistent across most cyclorrhaphan mt genomes except in three species: *Simosyrphus grandicornis* (Syrphidae) [12], *Spanicelyphus pilosus* (Celyphidae, present study) and *Stomoxys calcitrans* (Muscidae) [31], which have negative AT-skew on the J-strand (Table S1). Three other insect families: Philopteridae (Phthiraptera), Aleyrodidae (Hemiptera) and Braconidae (Hymenoptera) were found with positive GC-skew and negative AT-skew on the J-strand [56], and a strongly positive AT-skew on the J-strand was detected in Isoptera [57]. In insects, gene direction, replication and codon positions are all related to the degree of AT-skew, whereas reversals in replication orientation affects the degree of GC-skew [56].

### 2.3. Protein-Coding Genes and Codon Usage

The overall A + T content of the 13 PCGs in the five lauxanioid flies was between 74.3% (*Pachycerina decemlineata* and *Celyphus obtectus*) and 74.6% (*Cestrotus liui* and *Chamaemyia juncorum*). The A + T content of third codon positions (89.1−90.2%) was much higher than either the first (66.8−68.7%) or second codon positions (65.9–66.0%) (Table S1). The AT-skew was strongly negative at the second codon positions (from −0.396 to −0.394), while it was weakly negative at the first and third codon positions (from −0.108 to −0.071 and from −0.042 to −0.022, respectively), which results in a moderate negative AT-skew for the PCGs as a whole (from −0.166 to −0.147). On the other hand, the absence of significant CG-skew across the PCGs as a whole (from 0.000 to 0.031) masks strong skews at each codon position, as the strongly positive skew at the first codon position (from 0.227 to 0.253) is masked by strongly negative skews at the second and third codon positions (from −0.156 to −0.146 and from −0.208 to −0.038, respectively) (Table S1).

All the PCGs in the five lauxanioid flies used canonical start codons. For all species, the *ATP6*, *CO1*, *CO3*, *ND1*, *ND4* and *ND4L* genes started with ATG (Met), the *ATP8*, *ND2*, *ND3*, *ND5* and *ND6* genes started with ATT (Ile) (except *Celyphus obtectus* which used ATC (Ile) in *ATP8*, *ND3* and *ND5*). *Pachycerina decemlineata* and *Spanicelyphus pilosus* used ATG (Met) in *CO2*, while for other species it started with TCG (Ser). For *ND1*, *Cestrotus liui* and *Chamaemyia juncorum* started with ATT (Ile), while the remaining species used TTG (Leu). TCG (Ser) has been identified as the most frequent start codon for *CO1* in Cyclorrhapha [30], but ATG (Met) was used for *CO1* in all five lauxanioid flies (Table S2).

The stop codons most commonly used in the five lauxanioid flies are TAA (*ATP6*, *ATP8*, *CO2*, *CO3*, *ND2*, *ND4L* and *ND6*) (the exception is *Chamaemyia juncorum* with an incomplete stop codon T in *CO3*) or TAG (*CYTB*, *ND3*) (the exception is *Cestrotus liui* with TAA in *ND3*). For all species, the incomplete stop codon T was used in *CO1* (except *Cestrotus liui* and *Chamaemyia juncorum* which used TAA), *ND1*, *ND4* (*Cestrotus liui* used TAA) and *ND5* (*Cestrotus liui* used TAA, *Pachycerina decemlineata* used TAG) (Table S2).

**Figure 4:**
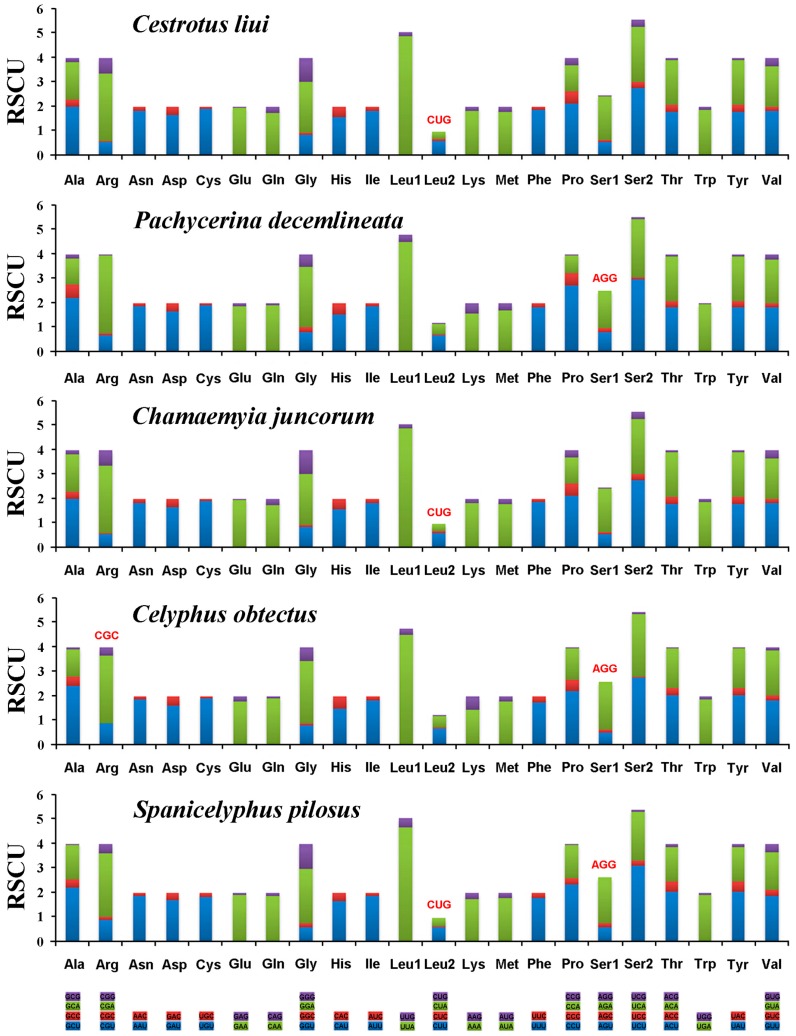
Relative synonymous codon usage (RSCU) in the five lauxanioid mt genomes. Codon families are provided on the *X*-axis. Stop codon is not given. Red codon, codon not present in the chain/genome.

A + T bias is also reflected in the relative codon usage by the PCGs. The amino acid frequencies excluding stop codons are similar amongst the different lauxanioid mitochondrial genomes (Figure 4). The most frequently used codons across all species were TAA (Leu), AAT (Ile), AAA (Lys), TAT (Tyr), ATT (Asn), and ATA (Met). The only exception was *Celyphus obtectus* where the proportion of TCC (Gly) was slightly higher than ATA (Met). Three codons were apparently not used in the mitochondrial PCGs of the five lauxanioid flies. GCG (Arg) was absent from the PCGs of *Celyphus obtectus*, CAG (Leu) was not present in the PCGs of *Cestrotus liui*, *Chamaemyia juncorum* and *Spanicelyphus pilosus*, and CCT (Ser) was absent from the PCGs of *Pachycerina decemlineata*, *Celyphus obtectus* and *Spanicelyphus pilosus* (Figure 4).

### 2.4. Intergenic Sequences

Two 18 bp intergenic sequences, highly conserved at the sequence level across the Cyclorrhapha, have been previously reported: between *ND1-tRNA^Ser^*^(*UCN*)^ and between *tRNA^Glu^*-*tRNA^Phe^* [30]. Similar to all the other Cyclorrhapha, the five lauxanioid flies also have these two conserved intergenic spacers. For the spacer between *ND1* and *tRNA^Ser^*^(*UCN*)^, all five lauxanioid flies have the 18 bp conserved sequence, but *Pachycerina decemlineata* has an additional 6 bp “TAAACT” at the 5′ end (N-strand), while *Cestrotus liui* and *Chamaemyia juncorum* have a redundant “A” at the 3′ end (N-strand). The conserved sequence region for this spacer in the Lauxanioidea is “TATBAAWWWWWWWTAGTA” (Figure S1A). For the spacer between *tRNA^Glu^* and *tRNA^Phe^*, *Cestrotus liui* and *Chamaemyia juncorum* have 22 and 25 bp intergenic sequences, respectively, while the basic 18 bp motif found across Cyclorrhapha is found in the other three species. The consensus sequence of this 18 bp motif is “ACTWAWWWWAWTTMWHWA” (Figure S1B).

**Figure 5:**
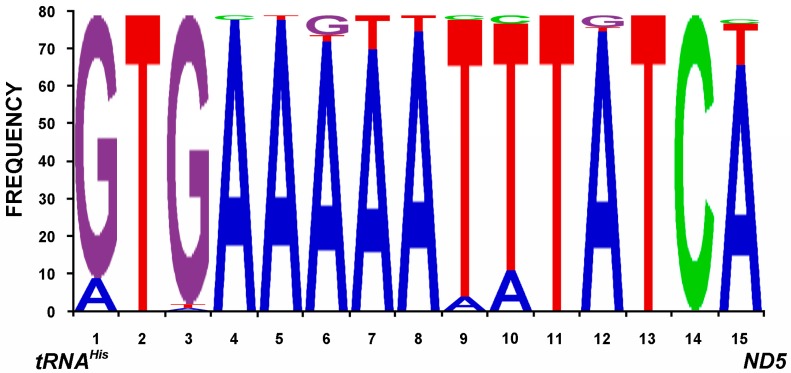
Conserved intergenic sequences between *tRNA^His^* and *ND5*, reversed sequences.

In addition to these two spacer regions, another non-coding region between *tRNA^His^* and *ND5* widely conserved in the Cyclorrhapha was detected in this study (Figure 5), which is often 15 bp in length with only three exceptions (14 bp in *Fergusonina taylori*, Fergusoninidae [14] and *Bactrocera minax*, Tephritidae [20] and 18 bp in *Ceratitis capitata*, Tephritidae [23]). The consensus sequence for this spacer amongst the lauxanioid flies was “GTGAAWWWTTTATCM” (Figure S1C). A non-coding region between *tRNA^His^* and *ND5* has been previously reported by Yang et al. [16], based on an analysis of 25 cyclorrhaphan mt genomes, with a 7 bp conserved motif. In the present study, a conserved 15 bp region was confirmed as present in all 79 available cyclorrhaphan mt genomes. More research is needed to determine the functions of conserved non-coding region in insect genomes, although the *ND1*-*tRNA^Ser^*^(*UCN*)^ spacer has been proposed as a likely translation termination site, mtTERM, that controls overexpression of the rRNA genes relative to the protein-coding genes [58,59].

### 2.5. Transfer RNAs

**Figure 6:**
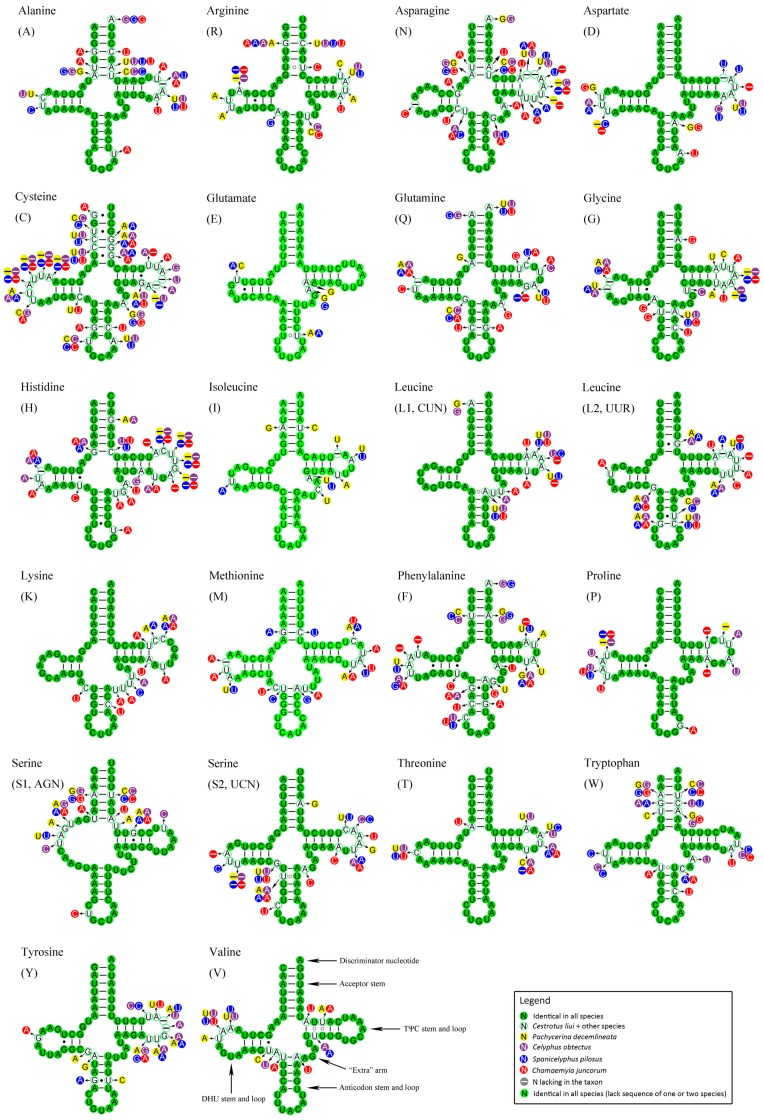
Putative secondary structures of tRNAs found in the five lauxanioid mt genomes. All tRNAs can be folded into the usual clover-leaf secondary structure. The tRNAs are labelled with the abbreviations of their corresponding amino acids. Inferred Watson–Crick bonds are illustrated by lines, whereas guanine–uracil (GU) bonds are illustrated by dots. The lauxanioid substitution pattern for each tRNA was modeled using as reference the structure determined for *Cestrotus liui*.

All 22 typical tRNAs found in the arthropod mt genomes were found in the three complete lauxanioid mt genomes, while 19 and 20 tRNAs were detected in the two partial genomes. Most tRNAs could be folded into the typical clover-leaf structure (Figure 6), while *tRNA^Ser^* ^(*AGN*)^ was an exception as it lacks a DHU arm, as has been observed in other metazoan mt genomes [60]. The combined length of all tRNAs was 1474 bp in *Cestrotus liui*, 1464 bp in *Pachycerina decemlineata* and 1467 bp in *Spanicelyphus pilosus*, which are medium-sized totals when compared with the mt genomes of other Cyclorrhapha for which total tRNA size ranges from 1450 bp (*Rutilia goerlingiana*, Tachinidae [14]) to 1499 bp (*Procecidochares utilis*, Tephritidae, Wu et al. unpublished).

A comparative analysis of the secondary structures of lauxanioid tRNAs was performed (Figure 6). The presence of mismatches in some tRNAs stems is a common molecular feature of arthropod mt genomes. The correct folding of paired structures is thought to be restored through post-transcriptional editing processes [61] or may represent unusual pairings [62]. Mismatches were also detected in the tRNAs of the five lauxanioid (Figure 6, Table S3). One U–U pair in the acceptor stem of *tRNA^Arg^* was conserved among four of five lauxanioid genomes, but was present as a U–C pair in *Chamaemyia juncorum*. The TΨC stem of *tRNA^Val^* had at least one U–U pair (all lauxanioid), and in one species (*Cestrotus liui*) had two pairs. The position of the U–U pair varied between second and third position in the stem in those species with only a single U–U pair.

In order to model the substitution patterns found in tRNAs, Negrisolo et al. [63] proposed two patterns: fully compensatory base changes (cbcs) (e.g., G–C to A–U) and hemi-cbc (e.g., G–U to A–U). Here we observed three more patterns: (1) reparative base changes (rbcs) which restore canonical pairs in a subset of taxa for a position where the majority of taxa lack canonical pairs (e.g., A–A to A–U on the anticodon stem of *tRNA^Leu^*^(*CUN*)^); (2) mirrored base changes (mbcs), a subset of fully cbcs for which the intermediate state is a non-canonical pair rather than a hemi-cbc pair (e.g., A–U to U–A as found in the acceptor stem of *tRNA^Ser^*^(*AGN*)^ in *Chamaemyia juncorum*); and (3) non-reparative base change (nrbcs), or substitutions from one non-canonical pair to another (e.g., U–U to U–C in the acceptor stem of *tRNA^Arg^* in *Chamaemyia juncorum*). All the stem-base changes observed in lauxanioid mt tRNA genes could be described by these five patterns, while the substitution changes on loops cannot be modeled properly due to the high level of variation among species.

The secondary structures of each tRNA genes across the Cyclorrhapha were compared (Figure S2). The TΨC loop was the most variable region, with the “extra” arm and TΨC stem ranking as the second and third most variable. Nucleotides were most conserved in the anticodon loop and DHU stem. Except for anticodon loop, the conservation of each stem was always higher than its corresponding loop. Most cyclorrhaphan tRNAs used the standard anticodon for each gene, but *tRNA^Asn^* in *Procecidochares utilis* (Tephritidae) (Wu et al. unpublished) was predicted to have the anticodon UUU, and *tRNA^Phe^* in *Liriomyza huidobrensis* (Agromyzidae) [15] used GAG as anticodon. Although the genetic code is nearly universal, more than ten variants have been described in metazoan mt genomes [63,64,65,66,67,68]. The above two patterns detected in cyclorrhapha mt genomes were unique amongst arthropods, while these variations on “wobble” position within the anticodon did not necessarily make changes to the genetic code.

**Figure 7:**
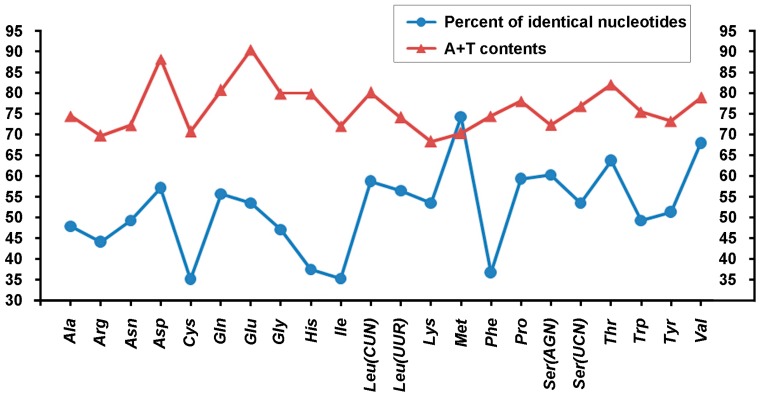
Nucleotides conservation and A + T contents of tRNAs in cyclorrhaphan mt genomes. Blue circle, percent of identical nucleotides; Red triangle, A + T contents.

The percent of identical nucleotides (%INUC) and the A + T content generated from alignments of tRNAs genes were calculated for the Cyclorrhapha (Figure 7). Amongst the Cyclorrhapha, three of the four most conserved tRNAs (%INUC ≥ 60), *tRNA^Met^* (74.3%), *tRNA^Ser^*^(*AGN*)^ (60.3%) and *tRNA^Thr^* (63.8%), are located on the J-strand, while only *tRNA^Val^* (68.1%) is encoded on the N-strand. Other tRNAs with high level of nucleotide conservation (55 ≤ %INUC < 60) include three J-strand tRNAs: *tRNA^Asp^*, *tRNA^Glu^*, *tRNA^Leu^*^(*UUR*)^, and two N-strand tRNAs: *tRNA^Leu^*^(*CUN*)^ and *tRNA^Pro^*. On the other hand, *tRNA^Cys^* (35.1%), which is encoded on the N-strand, is the least conserved tRNA. Other less conserved tRNAs (%INUC < 45) include *tRNA^His^* and *tRNA^Phe^* encoded on the N-strand, and *tRNA^Arg^* and *tRNA^Ile^* located on the J-strand. The nucleotide conservation pattern has been reported to have a remarkable J-strand bias in neuropterid tRNAs [62]. However, only a limited J-strand bias was observed in cyclorrhaphan tRNAs, with the J-strand tRNAs having %INUC ranging 35.3% to 74.3% (average 53.4%), while %INUC in the N-strand tRNAs was between 35.1% and 68.1% (average 50.1%). In contrast, the pattern of A + T% showed a modest N-strand bias. The two tRNAs with the highest A + T content were *tRNA^Glu^* (90.5%, encoded on the N-strand) and *tRNA^Asp^* (88.3%, encoded on the J-strand). Seven of the 11 tRNAs with low A + T content (<75%) are located on the J-strand, including the two tRNAs with the lowest A + T content, *tRNA^Arg^* (69.8%) and *tRNA^Lys^* (68.4%).

No relationships were observed between the %INUC of a tRNA, and its location relative to either the control region or the mtTERM site the *ND1*-*tRNA^Ser^*^(*UCN*)^ space discussed above for neuropterid tRNAs [63]. However, in the present analysis of cylorrhaphan tRNAs, those closest to the control region or either the *tRNA^Glu^*-*tRNA^Phe^* or *tRNA^His^*-*ND5* conserved intergenic spacers, were the least conserved tRNAs (*tRNA^His^* (37.5%), *tRNA^Ile^* (35.3%), *tRNA^Phe^* (36.8%)) (Figure 1 and Figure 7). Additionally, a similar trend was observed between %INUC and A + T content for cyclorrhaphan tRNAs (Figure 7) with Pearson Correlation Coefficient = 0.21, which indicated a weak positive correlation between them. In general, tRNAs with higher A + T content tended to be more conserved. The two exceptions were *tRNA^Met^* (exceedingly conserved, lower than average A + T) and *tRNA^Phe^* (very low conservation, average A + T) (Figure 7).

Analysis of tRNA conservation was extended to include all cyclorrhaphan superfamilies with more than two available complete mt genomes (Figure S3A). Different superfamilies exhibited different patterns of %INUC for their tRNAs. The most conserved tRNA in any superfamily was *tRNA^Leu^*^(*UUR*)^ in the Muscoidea (95.5%), while the lowest was *tRNA^Val^* (47.0%) in the Opomyzoidea. Across the Cyclorrhapha, a total of 16 tRNAs were found with %INUC ≥ 90 in a single superfamily: Ephydroidea (three tRNA genes), Lauxanioidea (two), Oestroidea (two), Tephritoidea (one) and Opomyzoidea (one). Muscoidea tRNAs had the highest level of conservation (from 75.7% to 95.5% with an average of 87.2%), followed by the Ephydroidea, Lauxanioidea and Opomyzoidea (averages of 83.4%, 81.8% and 80.4%, respectively). The Tephritoidea (from 64.3% to 94.1% with an average of 77.5%) and Oestroidea (from 57.4% to 91.7% with an average of 77.1%) had the least conserved tRNAs. The results of the A + T content analysis from these cyclorrhaphan superfamilies are summarized in Figure S3B. A similar pattern of A + T content was observed, *tRNA^Lys^* often had the lowest A + T content (in Tephritoidea, Opomyzoidea, Muscoidea, Oestroidea and Cyclorrhapha), while *tRNA^Glu^* often had the highest (in Ephydroidea, Opomyzoidea, Muscoidea, Oestroidea and Cyclorrhapha).

### 2.6. Ribosomal RNAs

Among the five lauxanioid mt genomes, the length of *lrRNA* ranges from 1312 bp (*Chamaemyia juncorum*) to 1334 bp (*Cestrotus liui*), and the lengths of *srRNAs* are 786 bp (*Cestrotus liui*), 788 bp (*Pachycerina decemlineata*) and 793 bp (*Spanicelyphus pilosus*) (the complete *srRNA* could not be amplified for the other two species). Both subunits of rRNA are encoded on the N-strand as in other insects. Unlike PCGs with functional annotation features like start and stop codons, it is difficult to determine the boundaries from rRNA gene sequences alone [29,69], therefore, the boundaries of flanking genes were used by assuming no overlapping or gaps located between adjacent genes. As in the inferred ancestral insect mt genome pattern, the *lrRNA* gene is located between *tRNA^Leu^*^(*CUN*)^ and *tRNA^Val^*, while the *srRNA* gene is between *tRNA^Val^* and the control region.

**Figure 8:**
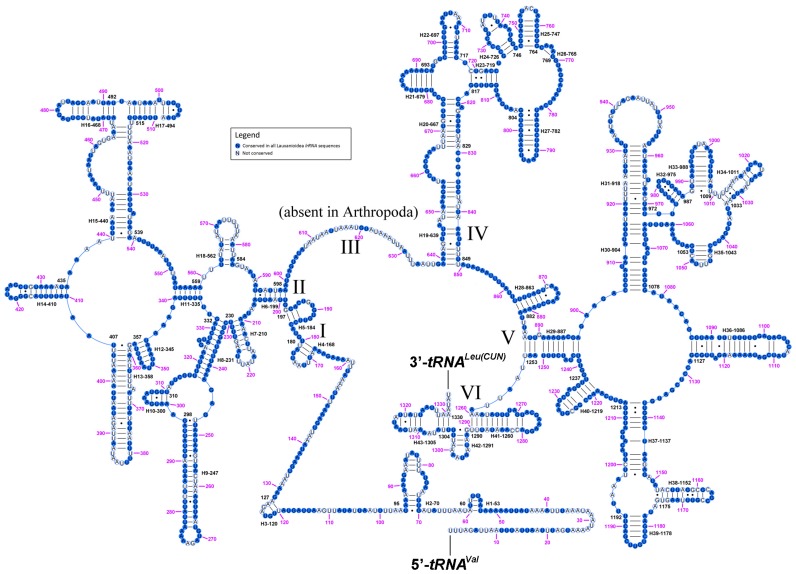
Predicted secondary structure of the *lrRNA* gene in *Cestrotus liui*. Filled circle, nucleotide conserved in five lauxanioid mt genomes; hollowed circle, nucleotide not conserved. Each helix is numbered progressively from 5′ to the 3′ end together with the first nucleotide belonging to the helix itself. Domains are labeled with Roman numerals. Inferred Watson–Crick bonds are illustrated by lines, GU bonds by dots.

**Figure 9:**
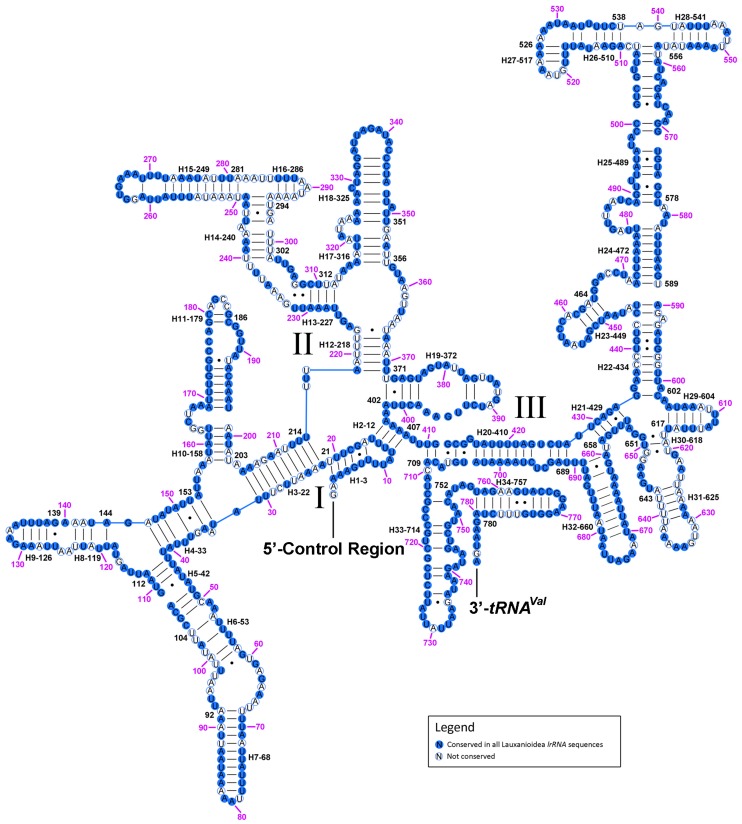
Predicted secondary structure of the *srRNA* gene in *Cestrotus liui*. Filled circle, nucleotide conserved in five lauxanioid mt genomes; hollowed circle, nucleotide not conserved. Each helix is numbered progressively from 5′ to the 3′ end together with the first nucleotide belonging to the helix itself. Domains are labeled with Roman numerals. Inferred Watson–Crick bonds are illustrated by lines, GU bonds by dots.

Secondary structures of both subunits of rRNA of *Cestrotus liui* mt genome were inferred using published rRNA secondary structures of *N. mamevi* [30] in Figure 8 and Figure 9, with nucleotides conserved among the five lauxanioid mt genomes shown in solid circles. The *lrRNA* had 43 helices in five structural domains (I–II, IV–VI, domain III is absent as in other insects). The multiple alignments of lauxanioid *lrRNAs* spanned 1350 positions and contained 974 conserved (with the same nucleotide in all five lauxanioid *lrRNAs*) (72.1%) and 376 variable positions (with at least one different nucleotide amongst five lauxanioid *lrRNAs*) (27.9%), respectively (Figure 8). The *srRNA* included three domains and 34 helices. The multiple alignment of lauxanioid *srRNAs* extended over 795 positions and contained 582 conserved (73.2%) and 213 variable sites (26.8%) (Figure 9). Secondary structures of all mt rRNAs from the Cyclorrhapha were inferred in Figures S4 and S5. Nucleotide conservation of the two rRNA genes was unevenly distributed among structural domains. Domains IV and V in *lrRNA* were more conserved than other domains, while the most conserved domain in *srRNA* was domain III (Figures S4 and S5).

Further analyses of the levels of nucleotide conservation and A + T content in the rRNAs were performed across the Cyclorrhapha as well as for each cyclorrhaphan superfamily with more than two available complete mt genomes (Figure S6). A similar positive correlation between %INUC and A + T content as that observed in tRNAs was detected from the rRNAs (Pearson Correlation Coefficient = 0.6 in *lrRNA* and 0.8 in *srRNA*). An extremely high %INUC was observed in the superfamily Ephydroidea, mainly due to all 14 available mt genomes for the Ephydroidea belonging to species from the same genus, *Drosophila*. The conservation of *lrRNA* amongst the Cyclorrhapha was 41.9%, while it was much lower for *srRNA* (31.0%). The Opomyzoidea had the highest A + T content in both rRNAs (83.2% in *lrRNA* and 80.7% in *srRNA*, respectively), and the rRNAs with the lowest A + T content belonged to the Tephritoidea (79.6% in *lrRNA* and 83.2% in *srRNA*, respectively). In general, the level of nucleotide conservation of *lrRNA*, as well as A + T content, were higher than those of *srRNA*, except for the Lauxanioidea where nucleotide conservation of *srRNAs* was slightly higher than that of *lrRNAs* (Figure S6).

### 2.7. The Control Region

**Figure 10:**
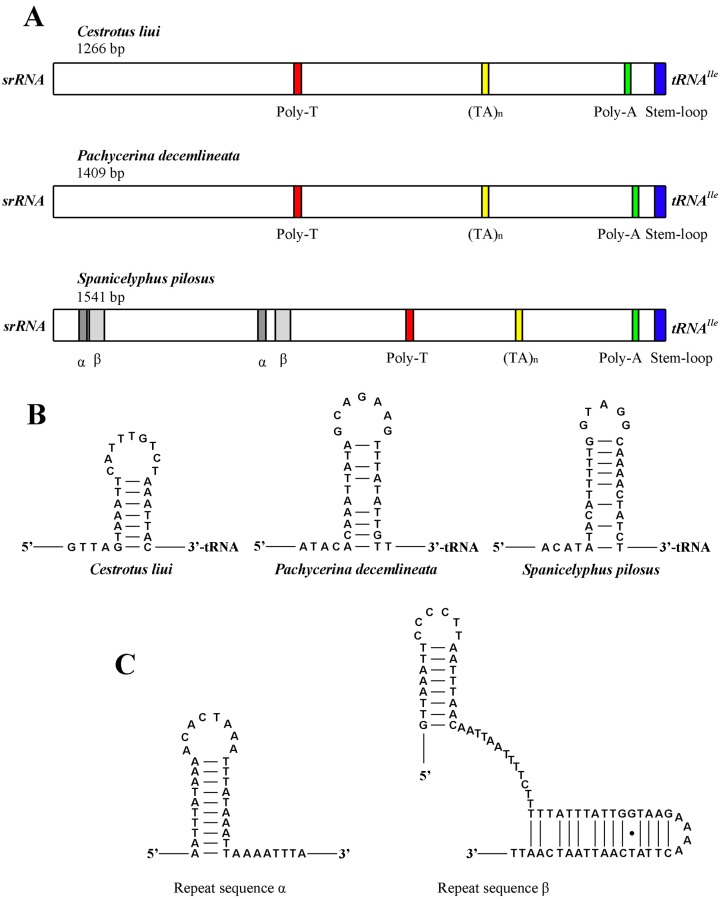
Predicted structure elements in the control region of *Cestrotus liui*, *Pachycerina decemlineata*, *Spanicelyphus pilosus*. (**A**) Control region structure of three species; (**B**) secondary structures of stem-loop structure at 3′-end of the control region; (**C**) secondary structures of repeat sequences of *Spanicelyphus pilosus*. Inferred Watson–Crick bonds are illustrated by lines, GU bonds by dots.

The control region is the longest non-coding region, located at the ancestral insect position between *srRNA* and *tRNA^Ile^*. Among the three complete lauxanioid mt genomes, the control regions range in size from 1266 bp (*Cestrotus liui*) to 1541 bp (*Spanicelyphus pilosus*). Four conserved structure elements were detected from all three completely sequenced control regions: (1) a poly-T stretch towards the middle of the control region (15 bp long in *Cestrotus liui*, 12 bp in *Pachycerina decemlineata* and 11 bp in *Spanicelyphus pilosus*); (2) a (TA)*_n_*-like stretch close to the poly-T stretch; (3) a poly-A stretch near the 3′-end of control region (13 bp in *Cestrotus liui*, 13 bp in *Pachycerina decemlineata* and 16 bp in *Spanicelyphus pilosus*); and, (4) a stem-loop structure at the 3′-end of the control region, that lacks both the 5′ “TATA” and 3′ “G(A)*_n_*T” consensus regions found in other insect mt control regions (Figure 10A,B).

Two long non-tandem macro repeats (72 bp and 36 bp, respectively) were found in the control region of *Spanicelyphus pilosus*: 5′-GTTAAATTCCCCTTAATTTAACAATTAATTTTCTTTTATTTATTGGTAAGAAAACTTATCAATTAATCAATT-3′ (from positions 199 to 270, and 584 to 655); and 5′-AATTTATAAAACACTAAATTTATAAATTAAAATTTA-3′ (from positions 162 to 197, and 547 to 582). Both macro repeats could be folded into stem-loop structures (Figure 10C). Additionally, several relatively short non-tandem repeats were detected from all three species, accompanied by a short (TA)*_n_*-like stretch. These (TA)*_n_*-like stretches can easily form stem-loop structures and might play roles in influence replication and transcription.

### 2.8. Phylogeny

Four datasets, varying by the inclusion and exclusion of different nucleotide classes, were used in the phylogenetic analysis. The four datasets are the P123 matrix (containing nucleotides of 13 PCGs) consisting of 10,977 residues, the P123R matrix (containing nucleotides of 13 PCGs, two rRNAs and 19 tRNAs) consisting of 13,903 residues, the P12 matrix (containing nucleotides of 13 PCGs but excluding the third codon sites) consisting of 7318 residues and the P12R matrix (containing nucleotides of 13 PCGs but excluding the third codon sites, two rRNAs and 19 tRNAs) consisting of 10,244 residues.

**Figure 11:**
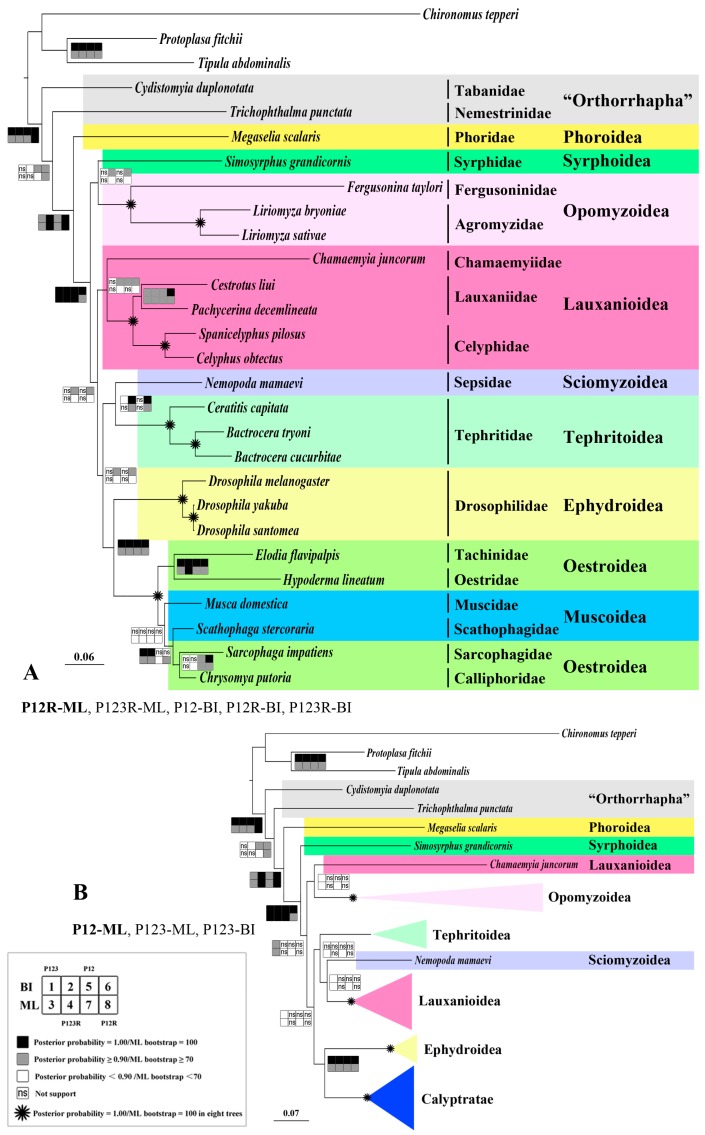
Phylogenetic trees of Brachycera families based on mt genome data. (**A**) Brachycera phylogeny obtained from the ML inferences based on P12R dataset, topology similar to P123R-ML, P12R-BI and P123R-BI; (**B**) Brachycera phylogeny obtained from the ML inferences based on P12 dataset, topology similar to P123-ML and P123BI. Cladogram of relationships with *Tipula abdominalis* (Tipulidae), *Chironomus tepperi* (Chironomidae) and *Protoplasa fitchii* (Tanyderidae) as outgroups. Squares at the nodes are Bayesian posterior probabilities for 1, 2, 5 and 6, ML bootstrap values for 3, 4, 7 and 8. Dataset of P123, 1 and 3, P123R, 2 and 4, P12, 5 and 7, P12R, 6 and 8. Black indicates posterior probabilities = 1.00 or ML bootstrap = 100; oblique lines indicates posterior probabilities ≥0.90, <1.00 or ML bootstrap ≥70, <100; white indicates posterior probabilities <0.90, ≥0.50 or ML bootstrap <70, ≥50; “ns” indicates posterior probabilities <0.50 or ML bootstrap <50, not supported, * indicates posterior probabilities = 1.00 or ML bootstrap = 100 in eight trees.

The phylogenetic trees inferred from both Bayesian and Maximum Likelihood (ML) analyses yield a consensus topology across the four datasets with the majority of nodes supported by all datasets and analyses (Figure 11A); all eight trees are shown in Table S7; discordant nodes will be discussed below. The monophyly of the Opomyzoidea, Tephritoidea, Ephydroidea and Calyptratae were consistently supported (posterior probability = 1.00 all datasets, ML bootstrap = 100 all datasets), as was the monophyly of the Brachycera (posterior probability = 1.00, ML bootstrap = 99/99/98/100 for the P123/P123R/P12/P12R datasets) and Cyclorrhapha (posterior probability = 0.99/1.00/0.99/1.00, ML bootstrap = 83/100/84/100). “Aschiza” was not monophyletic, and the Phoridae was the sister group of the remaining Cyclorrhapha (posterior probability = 1.00, ML bootstrap = 100/100/100/99), which is widely accepted by previous studies [48,50,70,71,72,73]. As has previously been found by Wiegmann et al. [51], (Ephydroidea + Calyptratae) formed a monophyletic group (posterior probability = 1.00, ML bootstrap = 83/93/78/96).

Similar to our previous analyses [30], the relationships between families in the Muscoidea (represented by the families Muscidae and Scathophagidae) and Oestroidea (represented by the families Oestridae, Tachinidae, Calliphoridae and Sarcophagidae) are highly discordant between the eight phylogenetic trees. Since it was specially analyzed by Ding et al. [33], two more muscoidean families are included here (Anthomyiidae and Fanniidae), relationships within the Calyptratae will not be discussed in this paper. Although contrary to some previous mt genome trees of the Diptera [7], the monophyly of the included “orthorrhaphan” taxa (Nemestrinidae and Tabanidae) was not supported by most of our analyses (except the BI-P123 (dataset P123 with Bayesian analysis), ML-P123and ML-P123R analyses) (Figure 11A); this is in accordance with more recent mt genome phylogenies of the lower Brachycera [74]. The paraphyly of “Orthorrhapha” has been widely recognized (e.g., [51,53,72]) and the Tabanidae (which belongs to the Tabanomorpha) has been considered to have a more basal position within the “Orthorrhapha” grade than the Nemestrinidae (typically assigned to the Asilomorpha but highly variable its phylogenetic position across different studies) [48,71].

The superfamily Lauxanioidea formed a monophyletic group in five of the eight analyses (posterior probability = ns/0.99/0.98/0.99, ML bootstrap = ns/51/ns/54) (Figure 11A). However, the other three analyses recovered a sister relationship between Chamaemyiidae and Opomyzoidea but without significant nodal support (posterior probability = 0.60/ns/ns/ns, ML bootstrap = 36/ns/36/ns) (Figure 11B). Amongst the superfamily Lauxanioidea, the monophyly of Celyphidae and its sister relationship to the Lauxaniidae were consistently and strongly supported (posterior probability = 1.00, ML bootstrap = 100), as was the monophyly of Lauxaniidae (posterior probability = 0.97/0.99/0.99/1.00, ML bootstrap = 71/73/78/86). This relationship (Chamaemyiidae + (Lauxaniidae + Celyphidae)), although relatively weakly supported here, supports the relationships proposed by McAlpine [49] for the superfamily. The inclusion of RNAs in the mitochondrial phylogenetic analysis has been shown to be beneficial in improving nodal confidence [12] or even stabilizing highly variable backbone relationships [5]. Here, the BI-P123, ML-P123 and ML-P12 datasets all failed to resolve the monophyly of the Lauxanioidea suggesting that the exclusion of RNAs is responsible for its non-monophyly in these analyses.

The backbone phylogeny of the Cyclorrhapha, however, was not well resolved in our analyses based on mt genome data. There are low nodal support values and/or conflict between datasets for many of the superfamily-level nodes. The clade (Sciomyzoidea + Tephritoidea) was only supported by datasets including RNAs (posterior probability = 0.6/1.0/ns/1.0, ML bootstrap = ns/86/ns/84), and was the weakly supported sister-group to the clade (Ephydroidea + Calyptratae) (posterior probability = ns/0.99/ns/0.99, ML bootstrap = ns/58/ns/58). The Lauxanioidea was sister to this derived set of superfamilies ((Sciomyzoidea + Tephritoidea) + (Ephydroidea + Calyptratae)); however, nodal support was weak (posterior probability = ns/0.99/ns/0.99, ML bootstrap = ns/50/ns/46) and again confined to datasets which include RNAs (Figure 11A). Schizophora including Syrphoidea was strongly monophyletic (posterior probability = 1.00, ML bootstrap = 100/100/100/99) (Figure 11A). This topology is also supported by Wiegmann et al. [51], which, however, did not recover a monophyletic Syrphoidea. Datasets which excluded RNAs indicated a different topology but with very low nodal supports for most of the main nodes (Figure 11B), as in Wiegmann et al.’s [51] analysis. However, the representative of Sciomyzoidea used here, Sepsidae, was more closely related to the Tephritoidea in Wiegmann et al.’s study [51], as opposed to being the sister of Lauxanioidea as in the present study.

The placement of Syrphoidea was weakly supported in both topologies. Either the Syrphoidea and the Opomyzoidea formed a clade (posterior probability = ns/0.98/ns/0.97, ML bootstrap = ns/53/ns/49), thus rendering the Schizophora non-monophyletic (Figure 11A), or the Syrphoidea was sister to the Schizophora (posterior probability = 0.96/ns/ns/ns, ML bootstrap = 72/ns/63/ns) (Figure 11B).

The relationships between acalyptrate fly families (i.e. Schiziphora excluding the Calyptratae) have been contentious and vary significantly between different phylogenetic analyses. The present study contributes to our efforts to understand these relationships while providing additional evidence that the inclusion of RNA genes in mt genome-based phylogenetic studies improves resolution [2]. A more comprehensive dataset using transcriptome or even genome data combined with morphological characters should be included in future studies.

## 3. Materials and Methods

### 3.1. Ethics Statement

No specific permits were required for the insects collected for this study. The specimen was collected by using sweeping method. The field studies did not involve endangered or protected species. The species herein studied are not included in the “List of Protected Animals in China”.

### 3.2. Sampling and DNA Extraction

The collection information for specimens used in this study is provided in Table S4. After collection, specimens were initially preserved in 95% ethanol in the field, and then transferred to −20 °C for the long-term storage upon the arrival at China Agricultural University (Beijng, China). Lauxaniidae specimens were examined and identified by Wenliang Li, Celyphidae specimens were examined and identified by Jinying Yang, Chamaemyiidae specimens were examined and identified by the first author Xuankun Li with a ZEISS Stemi 2000–c microscope (ZEISS, Jena, Germany). Whole genomic DNA was extracted from the thoracic muscle tissues using TIANamp Genomic DNA Kit (TIANGEN, Beijing, China). The quality of PCR products was assessed through electrophoresis in a 1% agarose gel and stained with Gold View (ACME, Beijing, China).

### 3.3. PCR Amplification and Sequencing

For each species, the mt genome was amplified by PCR in overlapping fragments using universal Diptera mt primers [7], and species-specific primers designed from sequenced fragments. All primers used in the present study are listed in Table S5. NEB Long Taq DNA polymerase (New England BioLabs, Ipswich, Suffolk, UK) was used to amplify PCR fragments.

PCR cycling consisted of an initial denaturation step at 95 °C for 30 s, followed by 40 cycles of denaturation at 95 °C for 10 s, annealing at 42–55 °C (depending on the primer pair used) for 50 s, elongation at 65 °C for 1 KB/min (depending on the size of target amplicon) (Table S1), and a final elongation step at 65 °C for 10 min. PCR products were evaluated by agarose gel electrophoreses.

All amplicons were sequenced in both directions using the BigDye Terminator Sequencing Kit (Applied Bio Systems, Waltham, MA, USA) and the ABI 3730XL Genetic Analyzer (PE Applied Biosystems, San Francisco, CA, USA) with two vector-specific primers and internal primers developed by primer walking.

### 3.4. Bioinformatic Analysis

Sequences were proofread and aligned into contigs in BioEdit version 7.0.5.3 [75]. After fully sequencing the mt genome, tRNA genes were identified with tRNAscan-SE 1.21 [76] with a cove cutoff score of 1 and the prediction of the genetic code followed the invertebrate mitochondrial DNA. tRNA genes not detected in this way were identified by comparison with multiple sequence alignments of the tRNAs in Cyclorrhapha. The secondary structures of tRNAs were also estimated by tRNAscan-SE Search Server v.1.21 [76], under the principles described by Lowe and Eddy [77].

Hand annotation method followed the procedures proposed by Cameron [3] plus modified quality control for partial stop codon by Li et al. [30]. The rRNA genes and the control region were identified by their boundaries with tRNA genes and comparison with other insect mt genomes.

The tRNAs’ secondary structures were identified by tRNAscan-SE Search Server v.1.21 [76], and the rRNAs were inferred using models for *Drosophila melanogaster* [26]. The secondary structures of RNAs are dependent on environmental conditions and, as presented, the RNAs’ secondary structures are primarily intended to show the substitution patterns within tRNAs as well as the conserved regions within rRNAs.

Nucleotide substitution rates, base composition and codon usage were analyzed with MEGA 5.0 [77]. Nucleotide compositional skew was measured using the following formula: AT-skew = (A − T)/(A + T) [78].

### 3.5. Phylogenetic Analysis

A total of 28 species of dipteran insects were used in phylogenetic analysis, including 25 brachycerans and three outgroup species from the “nematocera”: one species each from the families Tipulidae, Chironomidae and Tanyderidae. Details of the species used in this study are listed in Table 1.

Sequences of the 13 PCGs, two rRNAs and 19 tRNAs were used in phylogenetic analysis. Three tRNAs which were not available in all sampled dipterans were excluded: *tRNA^Ile^*, *tRNA^Gln^* and *tRNA^Met^*. The MAFFT algorithm in the TranslatorX online platform (http://translatorx.co.uk/) [79] under the L-INS-i strategy was utilized to align PCGs using codon-based multiple alignments and to toggle back to the nucleotide sequences. Before back-translating to nucleotides, poorly aligned sites were removed from the protein alignment using GBlocks v0.91b (http://molevol.cmima.csic.es/castresana/Gblocks_server.html) [80] as implemented in TranslatorX with default settings. MXSCARNA [81] was used to align tRNA genes based on the predicted secondary structures. The Muscle algorithm [82] as implemented in MEGA 5.0 [77] was performed to align the two rRNAs, and ambiguous positions in the alignment were filtered by hand based on the secondary structures predicted. Individual genes were concatenated using SequenceMatrix v1.7.8 [83]. We assembled four datasets for phylogenetic analysis: (1) all codon positions for the 13 PCGs (P123) 10,977 bp; (2) all codon positions for the 13 PCGs, plus the two rRNAs and 19 tRNAs (P123R) 13,903 bp; (3) the P123 dataset excluding third codon positions (P12) 7318 bp; and (4) the P123R dataset excluding third codon positions (P12R) 10,244 bp.

The optimal partition strategy and substitution models for each partition were selected by PartitionFinder v1.1.1 [84]. As the software requires the user to predefine partitions, we created input configuration files for the four datasets: (1) 39 partitions (three codon positions for each of the 13 PCGs) for P123; (2) 60 partitions (three codon positions for each of the 13 PCGs, 19 tRNA and two rRNA partitions) for P123R; (3) 26 partitions (two codon positions for each of the 13 PCGs) for P12; and (4) 47 partitions (two codon positions for each of the 13 PCGs, 19 tRNA and two rRNA partitions) for P123R. The best-fit partitioning schemes and models for ML and BI analyses of four datasets are obtained from the “greedy” algorithm calculated with “unlinked” branch lengths and the Bayesian information criterion (BIC) [85,86] (Table S6).

We performed Bayesian inference (BI) and maximum likelihood (ML) using the best-fit partitioning schemes recommended by PartitionFinder v1.1.1 (Table S6). MrBayes 3.2.2 was used to conduct Bayesian analysis [87]. Two simultaneous runs of two million generations each were conducted for each dataset, each run with one cold and three heated chains. Samples were drawn every 1,000 Markov chain Monte Carlo (MCMC) steps, with the first 25% discarded as burn-in. When the average standard deviation of split frequencies was below 0.01, we considered the stationarity as having been reached for that run. For ML analysis was performed by RAxML 8.0.0 [88] with 100 runs for searching an optimal tree and another 500 pseudo-replicates for the bootstrap analyses (random seed value 12345). Bootstrap values were mapped onto the optimal tree after searching using Sumtrees Version 4.0.0 [89].

## Figures and Tables

**Table 1:** Summary of mitochondrion (mt) genome sequences from Brachycera and three outgroups.

Family	Species	Published Information	Code	Length (bp)
Tipulidae	*Tipula abdominalis* ^#^	[11]	JN_861743	-
Chironomidae	*Chironomus tepperi* ^#^	[11]	NC_016167	15,652
Tanyderidae	*Protoplasa fitchii* ^#^	[11]	NC_016202	16,154
Nemestrinidae	*Trichophthalma punctate* *	[12]	NC_008755	16,396
Tabanidae	*Cydistomyia duplonotata* *	[12]	NC_008756	16,247
Phoridae	*Megaselia scalaris* *	[13]	NC_023794	15,599
Syrphidae	*Simosyrphus grandicornis* *	[12]	NC_008754	16,141
Fergusoninidae	*Fergusonina taylori* *	[14]	NC_016865	16,000
Agromyzidae	*Liriomyza bryoniae* *	[15]	NC_016713	16,183
	*Liriomyza huidobrensis*	[15]	NC_016716	16,236
	Liriomyza sativae *	[16]	NC_015926	15,551
	*Liriomyza trifolii*	[17]	NC_014283	16,141
Tephritidae	*Bactrocera carambolae*	[18]	NC_009772	15,915
	*Bactrocera correcta*	Wu et al. Unpublished	NC_018787	15,936
	Bactrocera cucurbitae *	Wu et al. Unpublished	NC_016056	15,825
	*Bactrocera dorsalis*	[19]	NC_008748	15,915
	*Bactrocera minax*	[20]	NC_014402	16,043
	*Bactrocera oleae*	[21]	NC_005333	15,815
	*Bactrocera papayae*	[18]	NC_009770	15,915
	*Bactrocera philippinensis*	[18]	NC_009771	15,915
	Bactrocera tryoni *	[22]	NC_014611	15,925
	Ceratitis capitata *	[23]	NC_000857	15,980
	*Procecidochares utilis*	Wu et al. Unpublished	NC_020463	15,922
Drosophilidae	*Drosophila ananassae*	[24]	BK006336 (Without CR)	-
	*Drosophila erecta*	[24]	BK006335 (Without CR)	-
	*Drosophila grimshawi*	[24]	BK006341 (Without CR)	-
	*Drosophila littoralis*	[25]	NC_011596	16,017
	Drosophila melanogaster *	[26]	NC_001709	19,517
	*Drosophila mojavensis*	[24]	BK006339 (Without CR)	-
	*Drosophila persimilis*	[24]	BK006337 (Without CR)	-
	*Drosophila pseudoobscura*	[27]	NC_018348 (Without CR)	-
	Drosophila santomea *	[28]	NC_023825	16,022
	*Drosophila sechellia*	[29]	NC_005780 (Without CR)	-
	*Drosophila simulans*	[29]	NC_005781 (Without CR)	-
	*Drosophila virilis*	[24]	BK006340 (Without CR)	-
	*Drosophila willistoni*	[24]	BK006338 (Without CR)	-
	Drosophila yakuba *	[10]	NC_001322	16,019
Sepsidae	Nemopoda mamaevi *	[30]	KM605250	15,878
Lauxaniidae	Cestrotus liui *	Present study	KX372559	16,171
	Pachycerina decemlineata *	Present study	KX372561	16,286
Chamaemyiidae	Chamaemyia juncorum *	Present study	KX372560	-
Celyphidae	Celyphus obtectus *	Present study	KX372558	-
	Spanicelyphus pilosus *	Present study	KX372562	16,426
Muscidae	*Haematobia irritans*	[31]	NC_007102	16,078
	Musca domestica *	[32]	NC_024855	16,108
	*Stomoxys calcitrans*	[31]	DQ533708	15,790
Anthomyiidae	*Delia platura*	[33]	KP01268	-
Fanniidae	*Euryomma* sp.	[33]	KP01269	-
Scathophagidae	Scathophaga stercoraria *	[32]	NC_024856	16,223
Calliphoridae	*Calliphora vicina*	[6]	NC_019639	16,112
	*Chrysomya albiceps*	[6]	NC_019631	15,491
	*Chrysomya bezziana*	[6]	NC_019632	15,236
	*Chrysomya megacephala*	[6]	NC_019633	15,273
	Chrysomya putoria *	[34]	NC_002697	15,837
	*Chrysomya rufifacies*	[6]	NC_019634	15,412
	*Chrysomya saffranea*	[6]	NC_019635	15,839
	*Protophormia terraenovae*	[6]	NC_019636	15,170
	*Cochliomyia hominivorax*	[35]	NC_002660	16,022
	*Lucilia cuprina*	[6]	NC_019573	15,952
	*Lucilia porphyrina*	[6]	NC_019637	15,877
	*Lucilia sericata*	[6]	NC_009733	15,945
	*Hemipyrellia ligurriens*	[6]	NC_019638	15,938
Polleniidae	*Pollenia rudis*	[6]	JX913761 (Partial Genome)	-
Oestridae	*Dermatobia hominis*	[36]	NC_006378	16,460
	Hypoderma lineatum *	[37]	NC_013932	16,354
Sarcophagidae	Sarcophaga impatiens *	[6]	NC_017605	15,169
	*Sarcophaga peregrina*	[38]	NC_023532	14,922
Tachinidae	Elodia flavipalpis *	[7]	NC_018118	14,932
	*Exorista sorbillans*	[39]	NC_014704	14,960
	*Rutilia goerlingiana*	[6]	NC_019640	15,331

“-” not available (unknown or incomplete data); “*” species used in phylogenetic analysis; “^#^” outgroup.

## Supplementary Materials

Supplementary materials can be found at www.mdpi.com/1422-0067/18/4/773/s1.
