# Supplementary material for: Mitochondrial Genomes Provide Insights into the Phylogeny of Lauxanioidea (Diptera: Cyclorrhapha)
Source: Int J Mol Sci. 2017 Apr 14;18(4):773. doi: 10.3390/ijms18040773 (PMC5412357; doi:10.3390/ijms18040773)

Mitochondrial Genomes Provide Insights into the Phylogeny of Lauxanioidea (Diptera: Cyclorrhapha)

Xuankun Li [1,†], Wenliang Li [2,†], Shuangmei Ding [1], Stephen L. Cameron [3], Meng Mao [4], Li Shi [5,*] and Ding Yang [1,*]

[1] Department of Entomology, China Agricultural University, Beijing 100193, China; xuankun.li@csiro.au (X.L.); shuangmeiding@163.com (S.D.)

[2] College of Forestry, Henan University of Science and Technology, Luoyang 471023, China; wenliangli@haust.edu.cn

[3] Department of Entomology, Purdue University, West Lafayette, IN 47907, USA; cameros@purdue.edu

[4] Department of Plant and Environmental Protection Science, University of Hawaii at Manoa, Honolulu, HI 96822, USA; mm663@uowmail.edu.au

[5] College of Agronomy, Inner Mongolia Agricultural University, Hohhot 010018, China

* Correspondences: lirui2003@imau.edu.cn (L.S.); dingyang@cau.edu.cn (D.Y.); Tel.: +86-0471-4317421 (L.S.); +86-010-62732999 (D.Y.)

† These authors contributed equally to this work.

Supporting Information

Table S1. Nucleotide composition of lauxanioidean mitochondrial genome

Feature	A+T%					AT-skew					GC-skew				
	Ces. l	*Pac. d*	*Cel. o*	*Spa. p*	*Cha. j*	*Ces. l*	*Pac. d*	*Cel. o*	*Spa. p*	*Cha. j*	*Ces. l*	*Pac. d*	*Cel. o*	*Spa. p*	*Cha. j*
Whole mitgenome	76.7	76.3	-	76.9	-	0.007	0.004	-	-0.009	-	-0.159	-0.159	-	-0.174	-
PCGs	74.6	74.3	74.3	74.5	74.6	-0.155	-0.166	-0.147	-0.157	-0.155	0.031	0.031	0.000	0.027	0.031
1st codon position	68.7	66.8	67.2	68.2	68.7	-0.105	-0.108	-0.071	-0.085	-0.106	0.251	0.251	0.227	0.253	0.249
2nd codon position	66.0	66.0	65.9	65.9	66.0	-0.394	-0.394	-0.396	-0.396	-0.394	-0.150	-0.150	-0.155	-0.146	-0.156
3rd codon position	89.8	90.2	90.0	89.1	89.8	-0.024	-0.042	-0.022	-0.033	-0.024	-0.038	-0.038	-0.208	-0.086	-0.038
tRNA genes	77.6	76.7	-	77.3	-	-0.008	-0.014	-	-0.014	-	0.147	0.147	-	0.114	-
lrRNA	81.8	82.0	82.2	82.0	82.0	-0.017	-0.002	0.022	-0.007	-0.068	0.308	0.308	0.274	0.330	0.359
srRNA	78.7	78.4	77.1	77.2	78.7	0.014	0.010	0.012	0.047	-0.060	0.283	0.283	0.263	0.246	0.333
Control region	86.8	84.7	-	88.7	-	0.016	-0.001	-	0.030	-	-0.218	-0.218	-	-0.274	-

Note: *Ces. l* indicates *Cestrotus liui*, *Pac. d* indicates *Pachycerina decemlineata*, *Cel. o* indicates *Celyphus obtectus*, *Spa. p* indicates *Spanicelyphus pilosus* and *Cha. j* indicates *Chamaemyia juncorum*. The A+T and G+C biases of protein-coding genes were calculated by AT-skew= [A-T]/[A+T] and GC-skew= [G-C]/[G+C], respectively.

Table S2 Start/stop codons in lauxanioidean mitochondrial genes

Taxon	ND2		CO2		CO1		ATP8		ATP6		CO3		ND3	
	ST	EN	ST	EN	ST	EN	ST	EN	ST	EN	ST	EN	ST	EN
Ces. l	ATT	TAA	TCG	TAA	ATG	TAA	ATT	TAA	ATG	TAA	ATG	TAA	ATT	TAA
Pac. d	ATT	TAA	ATG	TAA	ATG	T	ATT	TAA	ATG	TAA	ATG	TAA	ATT	TAG
Cel. o	ATT	TAA	TCG	TAA	ATG	T	ATC	TAA	ATG	TAA	ATG	TAA	ATC	TAG
Spa. p	ATT	TAA	ATG	TAA	ATG	T	ATT	TAA	ATG	TAA	ATG	TAA	ATT	TAG
Cha. j	ATT	TAA	TCG	TAA	ATG	TAA	ATT	TAA	ATG	TAA	ATG	T	ATT	TAG

Taxon	ND5		ND4		ND4L		ND6		CYTB		ND1	
	ST	EN	ST	EN	ST	EN	ST	EN	ST	EN	ST	EN
Ces. l	ATT	TAA	ATG	TAA	ATG	TAA	ATT	TAA	ATG	TAG	ATT	T
Pac. d	ATT	TAG	ATG	T	ATG	TAA	ATT	TAA	ATG	TAG	TTG	T
Cel. o	ATC	T	ATG	T	ATG	TAA	ATT	TAA	ATG	TAG	TTG	T
Spa. p	ATT	T	ATG	T	ATG	TAA	ATT	TAA	ATG	TAG	TTG	T
Cha. j	ATT	T	ATG	T	ATG	TAA	ATT	TAA	ATG	TAG	ATT	T

Note: *Ces. l* indicates *Cestrotus liui*, *Pac. d* indicates *Pachycerina decemlineata*, *Cel. o* indicates *Celyphus obtectus*, *Spa. p* indicates *Spanicelyphus pilosus* and *Cha. j* indicates *Chamaemyia juncorum*. ST, start codon; EN, end (stop) codon.

Table S3. Mismatches in tRNAs.

**Taxon**	**A**	**R**	**C**	**E**	**G**	**L2**	**S2**	**T**	**W**	**V**
Ces. l	-	U-U	-	U-U	-	A-A	U-U	-	U-U	2U-U
Pac. d	U-U	U-U	-	-	U-U	-	-	-	U-U	U-U
Cel. o	U-U	U-U	U-U	U-U	U-U	-	-	-	U-U	U-U
Spa. p	U-U	U-U	-	-	U-U	-	-	-	U-U	U-U
Cha. j	-	U-C	U-U	U-U	-	-	U-C	U-U	-	U-U

Note: *Ces. l* indicates *Cestrotus liui*, *Pac. d* indicates *Pachycerina decemlineata*, *Cel. o* indicates *Celyphus obtectus*, *Spa. p* indicates *Spanicelyphus pilosus* and *Cha. j* indicates *Chamaemyia juncorum*.

Table S4. Collection information of lauxanioidean flies newly sequenced in the present study.

**Taxon**	**Locality**	**Time**	**Collector**
Ces. l	Baihualing, Baoshan, Yunnan (N25°17′32.85″ E98°48′23.08″)	16, July 2013	Xuankun Li
Pac. d	Xiayadong, Yadong, Tibet (N27°25′31.99″ E88°55′45.60″)	19, July 2013	Xiaoyan Liu
Cel. o	Bawangling, Changjiang, Hainan (N19°07′21.84″ E109°04′45.64″)	4, April 2013	Ziqiang Sun
Spa. p	Wazidi, Mangshi, Yunnan (N24°26′12.68″ E98°35′16.55″)	5, July 2013	Xuankun Li
Cha. j	Yunwushan Mt., Guyuan, Ningxia (N36°14′20.57″ E106°23′12.51″)	4, Auguest 2013	Lei Zhang

Note: *Ces. l* indicates *Cestrotus liui*, *Pac. d* indicates *Pachycerina decemlineata*, *Cel. o* indicates *Celyphus obtectus*, *Spa. p* indicates *Spanicelyphus pilosus* and *Cha. j* indicates *Chamaemyia juncorum*.

Table S5. Primers used in this study.

Cestrotus liui

Number	Primer pairs (F/R)	Sequence (forward and reverse) 5'-3'
1	TM-J-206/N2-N-732	GCTAAATAAGCTAACAGGTTCAT/AAGGAAGTTTGGTTTAAACCTCC
2*	Ces-Z1-302/Ces-Z1-1976	CATGGTTAGGAGCTTGAATAGG/CGGTCAAAAGTAATACCAGTTGATC
3	TY-J-1460/C1-N-2191	TACAATCTATCGCCTAAACTTCAGCC/CCCGGTAAAATTAAAATATAAACTTC
4	C1-J-1751/TL2-N-3014	GGAGCTCCTGATATAGCATTCCC/TCCATTGCACTAATCTGCCATATTA
5	Cl-J-2183/C3-N-5460	CAACATTTATTTTGATTTTTTGG/TCAACAAAGTGTCAGTATCATGC
6	C3-J-5005/E-rev	CTCCAGCAATTGAATTAGGAGCTA/AGTGATAAGCCTCTTTTTGGCTTC
7	F-fw/N5-N-7707	CATTTGATTTGCATTCAAAAAGTATTG/AGGATGAGATGGATTAGGACTAG
8	H-fw/N4-N-8718	GAAACAGGAGTAGGAGCTGCTATAGC/GCTTATTCATCGGTTGCTCA
9*	Ces-Z2-8413/Ces-Z2-8893	CTATTTAATAAAGAAATTTCACC/CCTAAAGCTCATGTTGAAGCTCC
10	N4-J-8614/N4-N-9061	TGAGCAACAGAAGAATAAGC/ATCAACCTGAACGATTACAAG
11	N4-J-8944/I-rev	CAGGAGCTTCAACATGAGCTTTAGG/CTTATTTTTGATTTACAAGACCAATG
12	N4-J-9511/CB-N-11218	CCAAAATTGATAACCCTAAAGC/TCAGGTTGAATGTGAATTGG
13	CB-J-10933/N1-N-12051	TATGTTCTACCATGAGGACAAATATC/GATTTTGCTGAAGGTGAATCAGA
14*	Ces-Z3-11969/ Ces-Z3-12902	CAAAACCCCCACTTCTATATTCTAC/GATTGCGACCTCGATGTTGGATTAAG
15	LR-J-12883/LR-N-13398	CACCGGTTTGAACTCAGATC/CGCCTGTTTATCAAAAACAT
16	LR-J-12888/SR-N-14373	ACGCTGTTATCCCTAAAGTA/AATCCACGATGTACCTTACT
17*	Ces-Z4-14180/ Ces-Z4-14723	AAGAGCGACGGGCGATGTGTAC/GGCTAAATTTGTGCCAGCAGC
18	SR-J-14612/SR-N-14922	AGGGTATCTAATCCTAGTTT/AAGTTTTATTTTGGCTTA
19	SR-J-14646/N2-N-309	GCTGGCACAAATTAAATC/CTAAACCTATTCAAGTTCC

Pachycerina decemlineata

Number	Primer pairs (F/R)	Sequence (forward and reverse) 5’-3’
1	TM-J-206/N2-N-732	GCTAAATAAGCTAACAGGTTCAT/AAGGAAGTTTGGTTTAAACCTCC
2*	Pac-Z1-555/ Pac-Z1-1672	GAAGGGCTTAGATGAATTAATTC/CTAATCAATTTCCAAATCC
3	TY-J-1460/C1-N-2191	TACAATCTATCGCCTAAACTTCAGCC/CCCGGTAAAATTAAAATATAAACTTC
4	C1-J-1751/TL2-N-3014	GGAGCTCCTGATATAGCATTCCC/TCCATTGCACTAATCTGCCATATTA
5	Cl-J-2183/C3-N-5460	CAACATTTATTTTGATTTTTTGG/TCAACAAAGTGTCAGTATCATGC
6	C2-J-3530/A6-N-4493	AAGTTGATGGAACTCCTGGA/GTAAGTCGAACTGCTAATGT
7	C3-J-5005/E-rev	CTCCAGCAATTGAATTAGGAGCTA/AGTGATAAGCCTCTTTTTGGCTTC
8*	Pac-Z2-5945/Pac-Z2-7730	CATTTGATTTGCATTCAAAAAGTATTG/ AGGGTGAGATGGATTGGGGTTGG
8	H-fw/N4-N-8718	GAAACAGGAGTAGGAGCTGCTATAGC/GCTTATTCATCGGTTGCTCA
9	I-fw/N4-N-8924	CTATTTAATAAAGAAATTTCTCC/CCTAAAGCTCATGTTGAAGCTCC
10	N4-J-8614/N4-N-9061	TGAGCAACAGAAGAATAAGC/ATCAACCTGAACGATTACAAG
11	N4-J-8944/I-rev	CAGGAGCTTCAACATGAGCTTTAGG/CTTATTTTTGATTTACAAGACCAATG
12	N4-J-9511/CB-N-11218	CCAAAATTGATAACCCTAAAGC/TCAGGTTGAATGTGAATTGG
13	CB-J-10933/N1-N-12051	TATGTTCTACCATGAGGACAAATATC/GATTTTGCTGAAGGTGAATCAGA
14	N1-J-11891/16S-N-12855	ATCCTCCTCTTCTATATTCAAT/GATTGCGACCTCGATGTT
15	LR-J-12883/LR-N-13398	CACCGGTTTGAACTCAGATC/CGCCTGTTTATCAAAAACAT
16	LR-J-12888/SR-N-14373	ACGCTGTTATCCCTAAAGTA/AATCCACGATGTACCTTACT
17	SR-J-14233/SR-N-14756	AAGAGCGACGGGCGATGTGT/GACAAAATTCGTGCCAGCAGT
18	SR-J-14612/SR-N-14922	AGGGTATCTAATCCTAGTTT/AAGTTTTATTTTGGCTTA
19*	Pac-ZK-14698/Pac-ZK-280	GCTGGCACAAATTTTGCCAATA/CTAAACCTATTCATGCTCTTAATC

Celyphus obtectus

Number	Primer pairs (F/R)	Sequence (forward and reverse) 5’-3’
1	TM-J-206/N2-N-732	GCTAAATAAGCTAACAGGTTCAT/AAGGAAGTTTGGTTTAAACCTCC
2*	Cel-Z1-555/Cel-Z1-1672	GAAGGGCTCTCTTGAACTAATTC/CCAGTCAATTTCCAAATCC
3	TY-J-1460/C1-N-2191	TACAATCTATCGCCTAAACTTCAGCC/CCCGGTAAAATTAAAATATAAACTTC
4	C1-J-1751/TL2-N-3014	GGAGCTCCTGATATAGCATTCCC/TCCATTGCACTAATCTGCCATATTA
5*	Cel-Z1-1970/ Cel-Z1-3198	CAACATCTTTTCTGATTCTTTGG/GAATATTCATAGCTTCAATATC
6*	Cel-Z2-2561/ Cel-Z2-5272	GTAAATTTAACCTTCTTCCCTCAAC/TCTACAAAATGTCAATATCATGC
7	C3-J-5005/E-rev	CTCCAGCAATTGAATTAGGAGCTA/AGTGATAAGCCTCTTTTTGGCTTC
8*	Cel-Z3-5787/ Cel-Z3-7548	CATTTGATTTGCATTCAAAAAGTATTG/AGGATGAGATGGGTTAGGTTTGG
9	H-fw/N4-N-8718	GAAACAGGAGTAGGAGCTGCTATAGC/GCTTATTCATCGGTTGCTCA
10	I-fw/N4-N-8924	CTATTTAATAAAGAAATTTCTCC/CCTAAAGCTCATGTTGAAGCTCC
11	N4-J-8614/N4-N-9061	TGAGCAACAGAAGAATAAGC/ATCAACCTGAACGATTACAAG
12	N4-J-8944/I-rev	CAGGAGCTTCAACATGAGCTTTAGG/CTTATTTTTGATTTACAAGACCAATG
13*	Cel-Z4-9531/Cel-Z4-10829	GTTTTCGATTTCTTAC/GTTATTGCTAAAACAATAAAAGG
14	CB-J-10933/N1-N-12051	TATGTTCTACCATGAGGACAAATATC/GATTTTGCTGAAGGTGAATCAGA
15	N1-J-11891/16S-N-12855	ATCCTCCTCTTCTATATTCAAT/GATTGCGACCTCGATGTT
16	LR-J-12883/LR-N-13398	CACCGGTTTGAACTCAGATC/CGCCTGTTTATCAAAAACAT
17	LR-J-12888/SR-N-14373	ACGCTGTTATCCCTAAAGTA/AATCCACGATGTACCTTACT
18	SR-J-14233/SR-N-14756	AAGAGCGACGGGCGATGTGT/GACAAAATTCGTGCCAGCAGT
19	SR-J-14612/SR-N-14922	AGGGTATCTAATCCTAGTTT/AAGTTTTATTTTGGCTTA

Spanicelyphus pilosus

Number	Primer pairs (F/R)	Sequence (forward and reverse) 5’-3’
1	TM-J-206/N2-N-732	GCTAAATAAGCTAACAGGTTCAT/AAGGAAGTTTGGTTTAAACCTCC
2	N2-J-283/C1-N-1740	CATGACTAGGAACTTGAATAGG/AGAACTAAAGCAGGAGGTAA
3	TY-J-1460/C1-N-2191	TACAATCTATCGCCTAAACTTCAGCC/CCCGGTAAAATTAAAATATAAACTTC
4	C1-J-1751/TL2-N-3014	GGAGCTCCTGATATAGCATTCCC/TCCATTGCACTAATCTGCCATATTA
5	Cl-J-2183/C3-N-5460	CAACATTTATTTTGATTTTTTGG/TCAACAAAGTGTCAGTATCATGC
6	C3-J-5005/E-rev	CTCCAGCAATTGAATTAGGAGCTA/AGTGATAAGCCTCTTTTTGGCTTC
7	F-fw/N5-N-7707	CATTTGATTTGCATTCAAAAAGTATTG/AGGATGAGATGGATTAGGACTAG
8	H-fw/N4-N-8718	GAAACAGGAGTAGGAGCTGCTATAGC/GCTTATTCATCGGTTGCTCA
9	I-fw/N4-N-8924	CTATTTAATAAAGAAATTTCTCC/CCTAAAGCTCATGTTGAAGCTCC
10	N4-J-8614/N4-N-9061	TGAGCAACAGAAGAATAAGC/ATCAACCTGAACGATTACAAG
11	N4-J-8944/I-rev	CAGGAGCTTCAACATGAGCTTTAGG/CTTATTTTTGATTTACAAGACCAATG
12	N4-J-9511/CB-N-11218	CCAAAATTGATAACCCTAAAGC/TCAGGTTGAATGTGAATTGG
13	CB-J-10933/N1-N-12051	TATGTTCTACCATGAGGACAAATATC/GATTTTGCTGAAGGTGAATCAGA
14	N1-J-11891/16S-N-12855	ATCCTCCTCTTCTATATTCAAT/GATTGCGACCTCGATGTT
15	LR-J-12883/LR-N-13398	CACCGGTTTGAACTCAGATC/CGCCTGTTTATCAAAAACAT
16	LR-J-12888/SR-N-14373	ACGCTGTTATCCCTAAAGTA/AATCCACGATGTACCTTACT
17	SR-J-14233/SR-N-14756	AAGAGCGACGGGCGATGTGT/GACAAAATTCGTGCCAGCAGT
18	SR-J-14612/SR-N-14922	AGGGTATCTAATCCTAGTTT/AAGTTTTATTTTGGCTTA
19*	Spa-ZK-14703/Spa-ZK-261	CGCATCATTTGTGTAACCGCGGC/GCCGAAACTGTAATTATTGTTCCTG

Chamaemyia juncorum

Number	Primer pairs (F/R)	Sequence (forward and reverse) 5’-3’
1*	Cha-Z1-50/ Cha-Z1-618	CACTTATTATATTTTG/AAAGAAGTTTGATTTATAGCTCC
2*	Cha-Z2-453/ Cha-Z2-1603	CCCTTTCATTTTTGATTTCC/GATATAGCCTTTCCCCGAATAAA
3	TY-J-1460/C1-N-2191	TACAATCTATCGCCTAAACTTCAGCC/CCCGGTAAAATTAAAATATAAACTTC
4	C1-J-1751/TL2-N-3014	GGAGCTCCTGATATAGCATTCCC/TCCATTGCACTAATCTGCCATATTA
5*	Cha-Z3-2026/ Cha-Z3-5312	CAACATCTTTTTTGATTTTTTGG/TCTACAAAATGTCAATATCAAGC
6	C2-J-3530/A6-N-4493	AAGTTGATGGAACTCCTGGA/GTAAGTCGAACTGCTAATGT
7	C3-J-5005/E-rev	CTCCAGCAATTGAATTAGGAGCTA/AGTGATAAGCCTCTTTTTGGCTTC
8*	Cha-Z4-5822/Cha-Z4-7130	CATTTGATTTGCATTCAAAAAGTATTG/ATTTTGAGTTTGATTTAAAAAAAATTA
9	H-fw/N4-N-8718	GAAACAGGAGTAGGAGCTGCTATAGC/GCTTATTCATCGGTTGCTCA
10	I-fw/N4-N-8924	CTATTTAATAAAGAAATTTCTCC/CCTAAAGCTCATGTTGAAGCTCC
11	N4-J-8614/N4-N-9061	TGAGCAACAGAAGAATAAGC/ATCAACCTGAACGATTACAAG
12	N4-J-8944/I-rev	CAGGAGCTTCAACATGAGCTTTAGG/CTTATTTTTGATTTACAAGACCAATG
13*	Cha-Z5-9406/Cha-Z5-11104	CTAAAATTGAAAGACCTAAAGAAC/TCAGGTTGAATGTGGATAGG
14	CB-J-10933/N1-N-12051	TATGTTCTACCATGAGGACAAATATC/GATTTTGCTGAAGGTGAATCAGA
15	N1-J-11891/16S-N-12855	ATCCTCCTCTTCTATATTCAAT/GATTGCGACCTCGATGTT
16	LR-J-12883/LR-N-13398	CACCGGTTTGAACTCAGATC/CGCCTGTTTATCAAAAACAT
17*	Cha-Z6-12770/Cha-Z6-14228	ACGCTGTTATCCCTAAAGTA/CGATAATCCACGATGGATCTCAC
18	SR-J-14233/SR-N-14756	AAGAGCGACGGGCGATGTGT/GACAAAATTCGTGCCAGCAGT
19	SR-J-14612/SR-N-14922	AGGGTATCTAATCCTAGTTT/AAGTTTTATTTTGGCTTA

* Species-specific primers designed in this study.

Table S6. The best partitioning scheme selected by PartitionFinder for different dataset.

Dataset	Subset Partitions	Best Model
P123R-codon: 7 partitions (BI)	P1: (ATP6_pos1, CO1_pos1, CO2_pos1, CO3_pos1, CytB_pos1)	GTR+I+G
	P2: (ATP6_pos2, ATP8_pos2, CO1_pos2, CO2_pos2, CO3_pos2, CytB_pos2, ND1_pos2, ND2_pos2, ND3_pos2, ND4L_pos2, ND5_pos2, ND6_pos2)	GTR+I+G
	P3: (ATP6_pos3, ATP8_pos3, CO1_pos3, CO2_pos3, CO3_pos3, CytB_pos3, ND2_pos3, ND3_pos3, ND6_pos3)	HKY+I+G
	P4: (ATP8_pos1, Ala, Arg, Asn, Asp, Cys, Glu, Gly, His, Leu1, Leu2, Lys, ND1_pos1, ND2_pos1, ND3_pos1, ND4L_pos1, ND5_pos1, ND6_pos1, Phe, Pro, Ser1, Ser2, Thr, Trp, Tyr, Val, lrRNA, srRNA)	GTR+I+G
	P5: (ND1_pos3, ND4L_pos3, ND5_pos3)	HKY+G
	P6: (ND4_pos1, ND4_pos2)	GTR+I+G
	P7: (ND4_pos3)	HKY+I+G
P123R-codon: 8 partitions (ML)	P1: (ATP6_pos1, CO1_pos1, CO2_pos1, CO3_pos1, CytB_pos1)	GTR+I+G
	P2: (ATP6_pos2, ATP8_pos2, CO1_pos2, CO2_pos2, CO3_pos2, CytB_pos2, ND1_pos2, ND2_pos2, ND3_pos2, ND4L_pos2, ND5_pos2, ND6_pos2)	GTR+I+G

	P3: (ATP6_pos3, CO1_pos3, CO2_pos3, CO3_pos3, CytB_pos3, ND2_pos3, ND3_pos3, ND6_pos3)	GTR+I+G
	P4: (ATP8_pos1, ATP8_pos3, ND2_pos1, ND3_pos1, ND6_pos1)	GTR+I+G
	P5: (Ala, Arg, Asn, Asp, Cys, Glu, Gly, His, Leu1, Leu2, Lys, ND1_pos1, ND4L_pos1, ND5_pos1, Phe, Pro, Ser1, Ser2, Thr, Trp, Tyr, Val, lrRNA, srRNA)	GTR+I+G
	P6: (ND1_pos3, ND4L_pos3, ND5_pos3)	GTR+I+G
	P7: (ND4_pos1, ND4_pos2)	GTR+I+G
	P8: (ND4_pos3)	GTR+I+G
P123-codon: 7 partitions (BI)	P1: (ATP6_pos1, CO1_pos1, CO2_pos1, CO3_pos1, CytB_pos1)	GTR+I+G
	P2: (ATP6_pos2, ATP8_pos2, CO1_pos2, CO2_pos2, CO3_pos2, CytB_pos2, ND1_pos2, ND2_pos2, ND3_pos2, ND4L_pos2, ND5_pos2, ND6_pos2)	GTR+I+G
	P3: (ATP6_pos3, CO1_pos3, CO2_pos3, CO3_pos3, CytB_pos3, ND2_pos3, ND3_pos3, ND6_pos3)	HKY+I+G
	P4: (ATP8_pos1, ATP8_pos3, ND1_pos1, ND2_pos1, ND3_pos1, ND4L_pos1, ND5_pos1, ND6_pos1)	GTR+I+G
	P5: (ND1_pos3, ND4L_pos3, ND5_pos3)	HKY+G
	P6: (ND4_pos1, ND4_pos2)	GTR+I+G
	P7: (ND4_pos3)	HKY+I+G

P123-codon: 7 partitions (ML)	P1: (ATP6_pos1, CO1_pos1, CO2_pos1, CO3_pos1, CytB_pos1)	GTR+I+G
	P2: (ATP6_pos2, ATP8_pos2, CO1_pos2, CO2_pos2, CO3_pos2, CytB_pos2, ND1_pos2, ND2_pos2, ND3_pos2, ND4L_pos2, ND5_pos2, ND6_pos2)	GTR+I+G
	P3: (ATP6_pos3, CO1_pos3, CO2_pos3, CO3_pos3, CytB_pos3, ND2_pos3, ND3_pos3, ND6_pos3)	GTR+I+G
	P4: (ATP8_pos1, ATP8_pos3, ND1_pos1, ND2_pos1, ND3_pos1, ND4L_pos1, ND5_pos1, ND6_pos1)	GTR+I+G
	P5: (ND1_pos3, ND4L_pos3, ND5_pos3)	GTR+I+G
	P6: (ND4_pos1, ND4_pos2)	GTR+I+G
	P7: (ND4_pos3)	GTR+I+G
P12R-codon: 4 partitions (BI)	P1: (ATP6_pos1, CO1_pos1, CO2_pos1, CO3_pos1, CytB_pos1)	GTR+I+G
	P2: (ATP6_pos2, ATP8_pos2, CO1_pos2, CO2_pos2, CO3_pos2, CytB_pos2, ND1_pos2, ND2_pos2, ND3_pos2, ND4L_pos2, ND4_pos2, ND5_pos2, ND6_pos2)	GTR+I+G
	P3: (ATP8_pos1, Asn, Asp, Glu, ND2_pos1, ND3_pos1, ND6_pos1)	GTR+I+G
	P4: (Ala, Arg, Cys, Gly, His, Leu1, Leu2, Lys, ND1_pos1, ND4L_pos1, ND4_pos1, ND5_pos1, Phe,	GTR+I+G

	Pro, Ser1, Ser2, Thr, Trp, Tyr, Val, lrRNA, srRNA)	
P12R-codon: 3 partitions (ML)	P1: (ATP6_pos1, CO1_pos1, CO2_pos1, CO3_pos1, CytB_pos1)	GTR+I+G
	P2: (ATP6_pos2, ATP8_pos2, CO1_pos2, CO2_pos2, CO3_pos2, CytB_pos2, ND1_pos2, ND2_pos2, ND3_pos2, ND4L_pos2, ND4_pos2, ND5_pos2, ND6_pos2)	GTR+I+G
	P3: (ATP8_pos1, Ala, Arg, Asn, Asp, Cys, Glu, Gly, His, Leu1, Leu2, Lys, ND1_pos1, ND2_pos1, ND3_pos1, ND4L_pos1, ND4_pos1, ND5_pos1, ND6_pos1, Phe, Pro, Ser1, Ser2, Thr, Trp, Tyr, Val, lrRNA, srRNA)	GTR+I+G
P12-codon: 4 partitions (BI)	P1: (ATP6_pos1, CO1_pos1, CO2_pos1, CO3_pos1, CytB_pos1)	GTR+I+G
	P2: (ATP6_pos2, ATP8_pos1, ATP8_pos2, CO1_pos2, CO2_pos2, CO3_pos2, CytB_pos2, ND1_pos2, ND2_pos2, ND3_pos2, ND4L_pos2, ND5_pos2, ND6_pos2)	GTR+I+G
	P3: (ND1_pos1, ND2_pos1, ND3_pos1, ND4L_pos1, ND5_pos1, ND6_pos1)	GTR+I+G
	P4: (ND4_pos1, ND4_pos2)	GTR+I+G
P12-codon:	P1: (ATP6_pos1, CO1_pos1, CO2_pos1, CO3_pos1,	GTR+I+G

4 partitions (ML)	CytB_pos1)	
	P2: (ATP6_pos2, ATP8_pos2, CO1_pos2, CO2_pos2, CO3_pos2, CytB_pos2, ND1_pos2, ND2_pos2, ND3_pos2, ND4L_pos2, ND5_pos2, ND6_pos2)	GTR+I+G
	P3: (ATP8_pos1, ND1_pos1, ND2_pos1, ND3_pos1, ND4L_pos1, ND5_pos1, ND6_pos1)	GTR+I+G
	P4: (ND4_pos1, ND4_pos2)	GTR+I+G
P123R-Al-codon: 7 partitions (BI)	P1: (ATP6_pos1, CO1_pos1, CO2_pos1, CO3_pos1, CytB_pos1)	GTR+I+G
	P2: (ATP6_pos2, ATP8_pos2, CO1_pos2, CO2_pos2, CO3_pos2, CytB_pos2, ND1_pos2, ND2_pos2, ND3_pos2, ND4L_pos2, ND5_pos2, ND6_pos2)	GTR+I+G
	P3: (ATP6_pos3, ATP8_pos3, CO1_pos3, CO2_pos3, CO3_pos3, CytB_pos3, ND2_pos3, ND3_pos3, ND6_pos3)	HKY+I+G
	P4: (ATP8_pos1, Ala, Arg, Asn, Asp, Cys, Glu, Gly, His, Leu1, Leu2, Lys, ND1_pos1, ND2_pos1, ND3_pos1, ND4L_pos1, ND5_pos1, ND6_pos1, Phe, Pro, Ser1, Ser2, Thr, Trp, Tyr, Val, lrRNA, srRNA)	GTR+I+G
	P5: (ND1_pos3, ND4L_pos3, ND5_pos3)	HKY+G
	P6: (ND4_pos1, ND4_pos2)	GTR+I+G
	P7: (ND4_pos3)	HKY+I+G

P123R-Al-codon: 8 partitions (ML)	P1: (ATP6_pos1, CO1_pos1, CO2_pos1, CO3_pos1, CytB_pos1)	GTR+I+G
	P2: (ATP6_pos2, ATP8_pos2, CO1_pos2, CO2_pos2, CO3_pos2, CytB_pos2, ND1_pos2, ND2_pos2, ND3_pos2, ND4L_pos2, ND5_pos2, ND6_pos2)	GTR+I+G
	P3: (ATP6_pos3, CO1_pos3, CO2_pos3, CO3_pos3, CytB_pos3, ND2_pos3, ND3_pos3, ND6_pos3)	GTR+I+G
	P4: (ATP8_pos1, ATP8_pos3, ND2_pos1, ND3_pos1, ND6_pos1)	GTR+I+G
	P5: (Ala, Arg, Asn, Asp, Cys, Glu, Gly, His, Leu1, Leu2, Lys, ND1_pos1, ND4L_pos1, ND5_pos1, Phe, Pro, Ser1, Ser2, Thr, Trp, Tyr, Val, lrRNA, srRNA)	GTR+I+G
	P6: (ND1_pos3, ND4L_pos3, ND5_pos3)	GTR+I+G
	P7: (ND4_pos1, ND4_pos2)	GTR+I+G
	P8: (ND4_pos3)	GTR+I+G
P123-Al-codon: 7 partitions (BI)	P1: (ATP6_pos1, CO1_pos1, CO2_pos1, CO3_pos1, CytB_pos1)	GTR+I+G
	P2: (ATP6_pos2, ATP8_pos2, CO1_pos2, CO2_pos2, CO3_pos2, CytB_pos2, ND1_pos2, ND2_pos2, ND3_pos2, ND4L_pos2, ND5_pos2, ND6_pos2)	GTR+I+G
	P3: (ATP6_pos3, CO1_pos3, CO2_pos3, CO3_pos3, CytB_pos3, ND2_pos3, ND3_pos3, ND6_pos3)	HKY+I+G

	P4: (ATP8_pos1, ATP8_pos3, ND1_pos1, ND2_pos1, ND3_pos1, ND4L_pos1, ND5_pos1, ND6_pos1)	GTR+I+G
	P5: (ND1_pos3, ND4L_pos3, ND5_pos3)	HKY+G
	P6: (ND4_pos1, ND4_pos2)	GTR+I+G
	P7: (ND4_pos3)	HKY+I+G
P123-Al-codon: 7 partitions (ML)	P1: (ATP6_pos1, CO1_pos1, CO2_pos1, CO3_pos1, CytB_pos1)	GTR+I+G
	P2: (ATP6_pos2, ATP8_pos2, CO1_pos2, CO2_pos2, CO3_pos2, CytB_pos2, ND1_pos2, ND2_pos2, ND3_pos2, ND4L_pos2, ND5_pos2, ND6_pos2)	GTR+I+G
	P3: (ATP6_pos3, CO1_pos3, CO2_pos3, CO3_pos3, CytB_pos3, ND2_pos3, ND3_pos3, ND6_pos3)	GTR+I+G
	P4: (ATP8_pos1, ATP8_pos3, ND1_pos1, ND2_pos1, ND3_pos1, ND4L_pos1, ND5_pos1, ND6_pos1)	GTR+I+G
	P5: (ND1_pos3, ND4L_pos3, ND5_pos3)	GTR+I+G
	P6: (ND4_pos1, ND4_pos2)	GTR+I+G
	P7: (ND4_pos3)	GTR+I+G
P123R-gene: 7 partitions (BI)	P1: (ATP6, CO1, CO2, CO3, CytB, ND3)	GTR+I+G
	P2: (ATP8, ND2, ND6)	GTR+I+G
	P3: (ND1, ND4L, ND5)	GTR+I+G
	P4: (ND4)	GTR+I+G
	P5: (Ala, Arg, Asn, Asp, Cys, Glu, Gly, His, Leu1,	GTR+I+G

	Leu2, Lys, Phe, Pro, Ser1, Ser2, Thr, Trp, Tyr, Val, lrRNA, srRNA)	
P123R-gene:	P1: (ATP6, CO1, CO2, CO3, CytB, ND3)	GTR+I+G
7 partitions (ML)	P2: (ATP8, ND2, ND6)	GTR+I+G
	P3: (ND1, ND4L, ND5)	GTR+I+G
	P4: (ND4)	GTR+I+G
	P5: (Ala, Arg, Asn, Asp, Cys, Glu, Gly, His, Leu1, Leu2, Lys, Phe, Pro, Ser1, Ser2, Thr, Trp, Tyr, Val, lrRNA, srRNA)	GTR+I+G
P123-gene:	P1: (ATP6, CO1, CO2, CO3, CytB)	GTR+I+G
4 partitions (BI)	P2: (ATP8, ND2, ND3, ND6)	GTR+I+G
	P3: (ND1, ND4L, ND5)	GTR+I+G
	P4: (ND4)	GTR+I+G
P123-gene:	P1: (ATP6, CO1, CO2, CO3, CytB)	GTR+I+G
4 partitions (ML)	P2: (ATP8, ND2, ND3, ND6)	GTR+I+G
	P3: (ND1, ND4L, ND5)	GTR+I+G
	P4: (ND4)	GTR+I+G
P12R-gene:	P1: (ATP6, CO1, CO2, CO3, CytB)	GTR+I+G
4 partitions (BI)	P2: (ATP8, Asn, Asp, Glu, ND2, ND3, ND6)	GTR+I+G
	P3: (ND1, ND4, ND4L, ND5)	GTR+I+G
	P4: (Ala, Arg, Cys, Gly, His, Leu1, Leu2, Lys, Phe, Pro, Ser1, Ser2, Thr, Trp, Tyr, Val, lrRNA, srRNA)	GTR+I+G

P12R-gene:	P1: (ATP6, CO1, CO2, CO3, CytB)	GTR+I+G
4 partitions (ML)	P2: (ATP8, Glu, ND2, ND3, ND6)	GTR+I+G
	P3: (ND1, ND4, ND4L, ND5)	GTR+I+G
	P4: (Ala, Arg, Asn, Asp, Cys, Gly, His, Leu1, Leu2, Lys, Phe, Pro, Ser1, Ser2, Thr, Trp, Tyr, Val, lrRNA, srRNA)	GTR+I+G
P12-gene:	P1: (ATP6, CO1, CO2, CO3, CytB)	GTR+I+G
4 partitions (BI)	P2: (ATP8, ND1, ND2, ND3, ND4L, ND5, ND6)	GTR+I+G
	P3: (ND4)	GTR+I+G
P12-gene:	P1: (ATP6, CO1, CO2, CO3, CytB)	GTR+I+G
3 partitions (ML)	P2: (ATP8, ND1, ND2, ND3, ND4L, ND5, ND6)	GTR+I+G
	P3: (ND4)	GTR+I+G
P123R-Al-gene:	P1: (ATP6, ATP8, ND2, ND3, ND6)	GTR+I+G
7 partitions (BI)	P2: (CO1, CO2, CO3, CytB)	GTR+I+G
	P3: (ND1, ND4L, ND5)	GTR+I+G
	P4: (ND4)	GTR+I+G
	P5: (Ala, Arg, Asn, Asp, Cys, Glu, Gly, His, Leu1, Leu2, Lys, Phe, Pro, Ser1, Ser2, Thr, Trp, Tyr, Val, lrRNA, srRNA)	GTR+I+G
P123R-Al-gene:	P1: (ATP6, ATP8, ND2, ND3, ND6)	GTR+I+G
7 partitions (ML)	P2: (CO1, CO2, CO3, CytB)	GTR+I+G
	P3: (ND1, ND4L, ND5)	GTR+I+G

	P4: (ND4)	GTR+I+G
	P5: (Ala, Arg, Asn, Asp, Cys, Glu, Gly, His, Leu1, Leu2, Lys, Phe, Pro, Ser1, Ser2, Thr, Trp, Tyr, Val, lrRNA, srRNA)	GTR+I+G
P123-Al-gene:	P1: (ATP6, CO1, CO2, CO3, CytB)	GTR+I+G
4 partitions (BI)	P2: (ATP8, ND2, ND3, ND6)	GTR+I+G
	P3: (ND1, ND4L, ND5)	GTR+I+G
	P4: (ND4)	GTR+I+G
P123-Al-gene:	P1: (ATP6, CO1, CO2, CO3, CytB)	GTR+I+G
4 partitions (ML)	P2: (ATP8, ND2, ND3, ND6)	GTR+I+G
	P3: (ND1, ND4L, ND5)	GTR+I+G
	P4: (ND4)	GTR+I+G

Table S7. Phylogenetic trees generated from different datasets and methods.

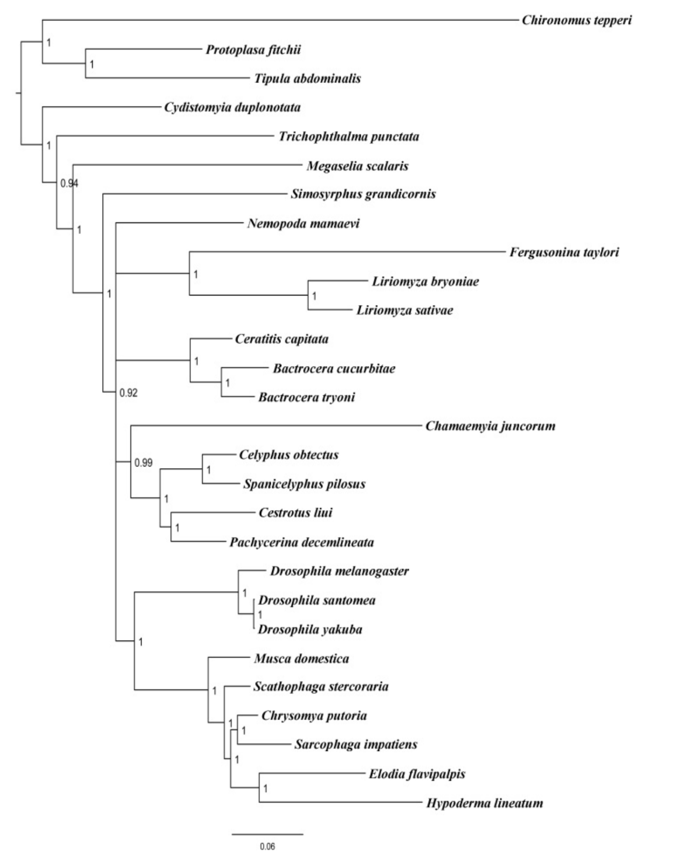

P12_BI

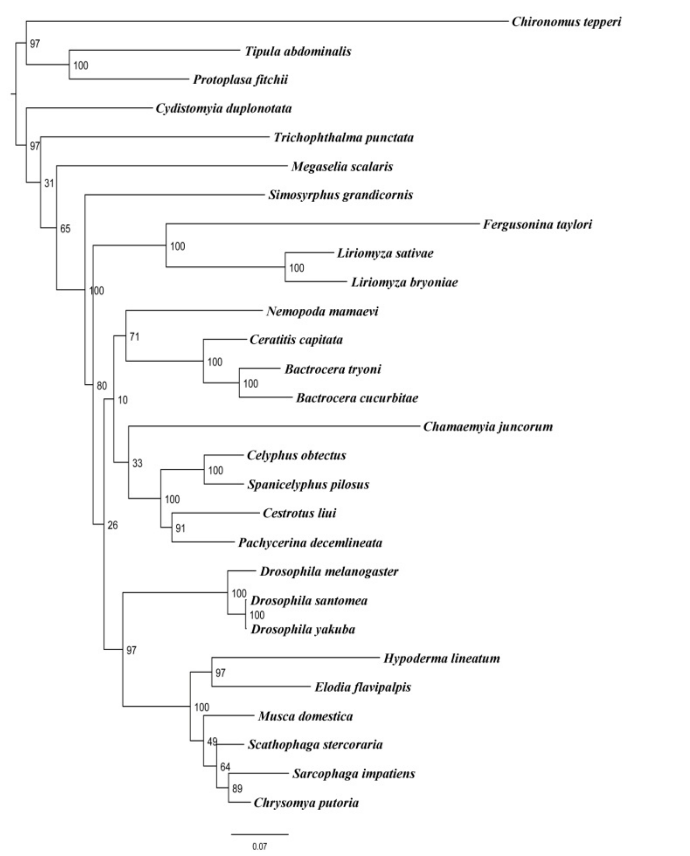

P12_ML

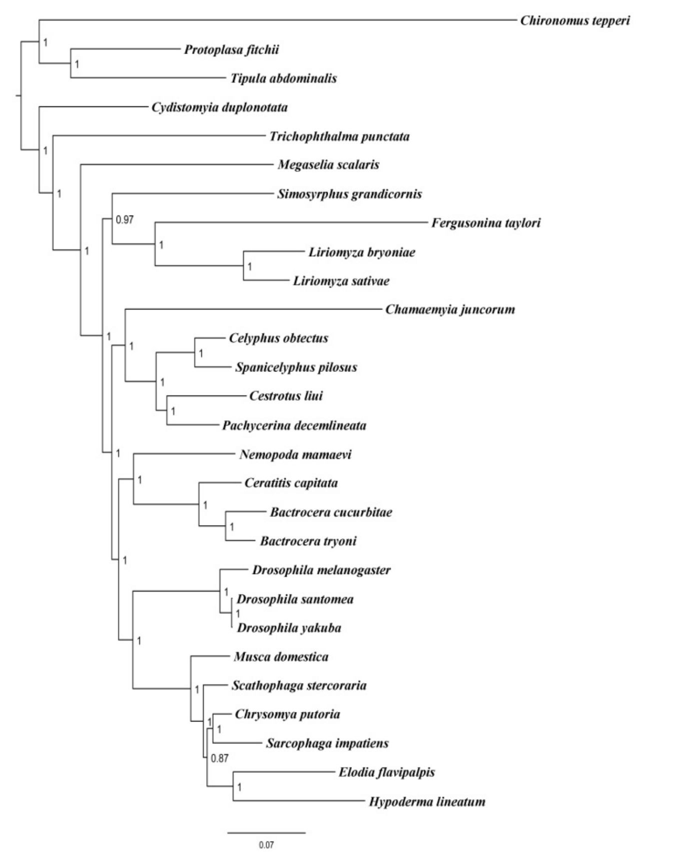

P12R_BI

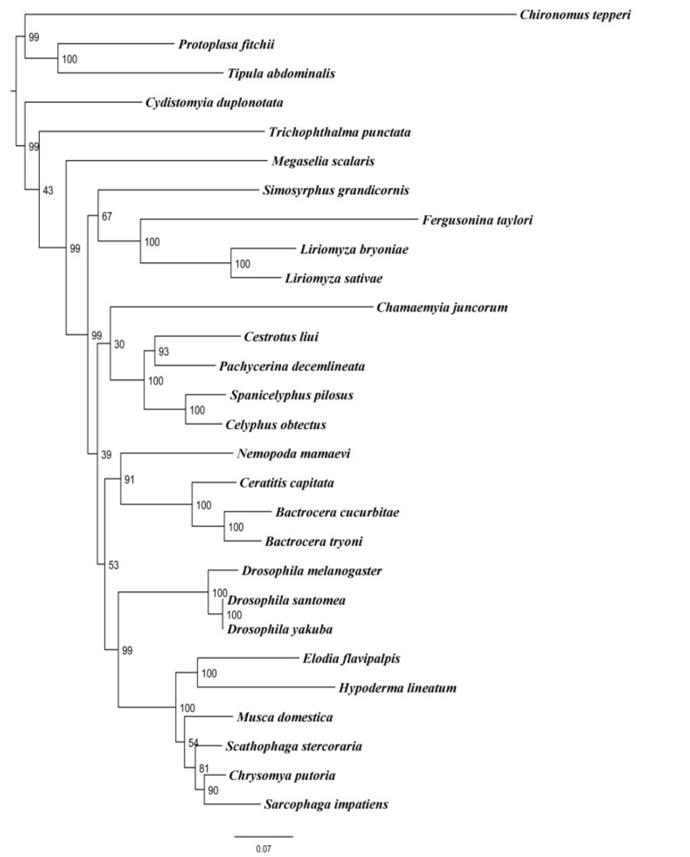

P12R_ML

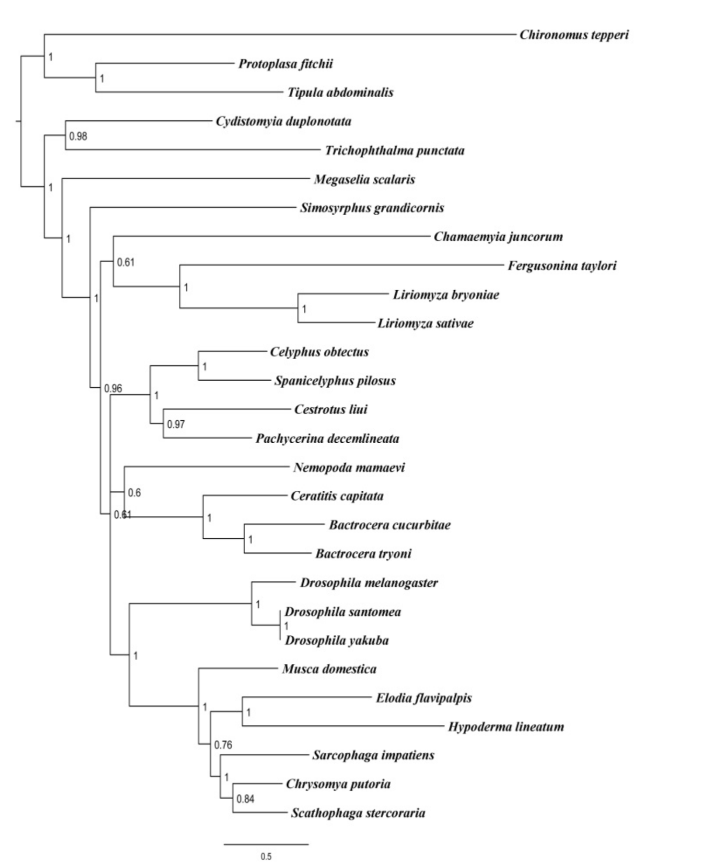

P123_BI

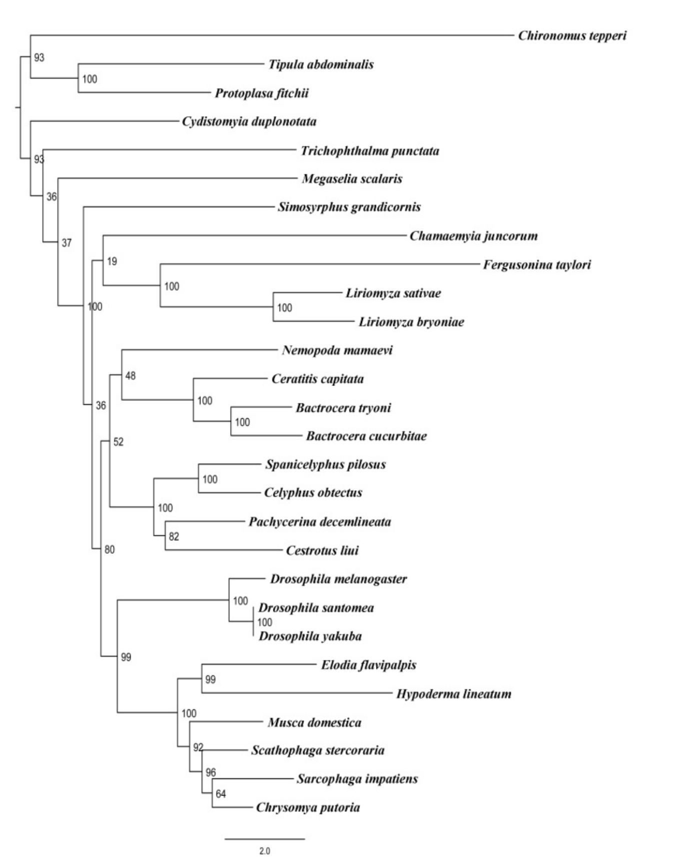

P123_ML

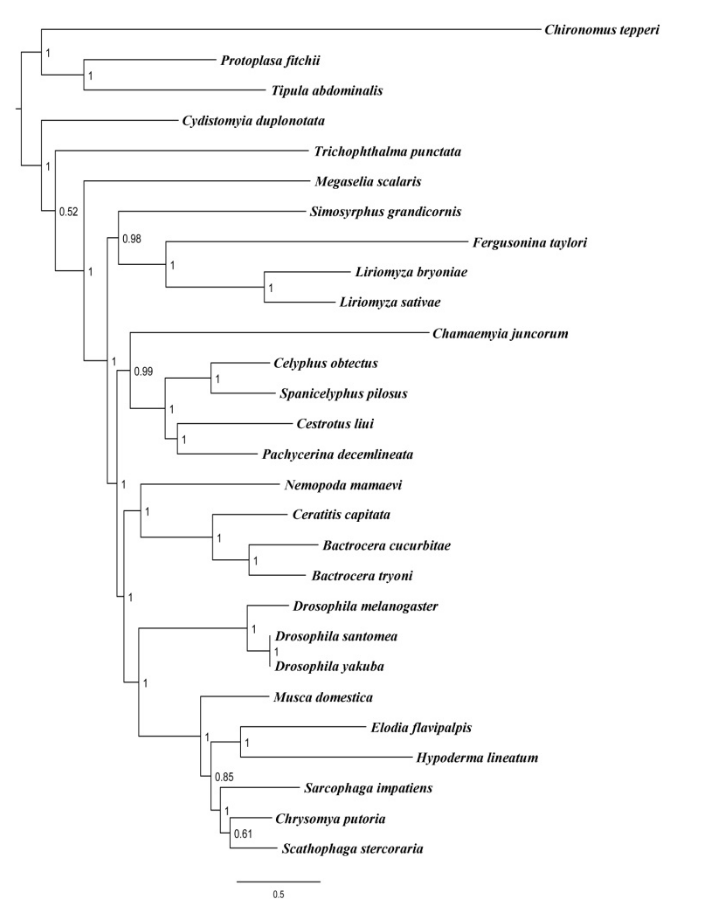

P123R_BI

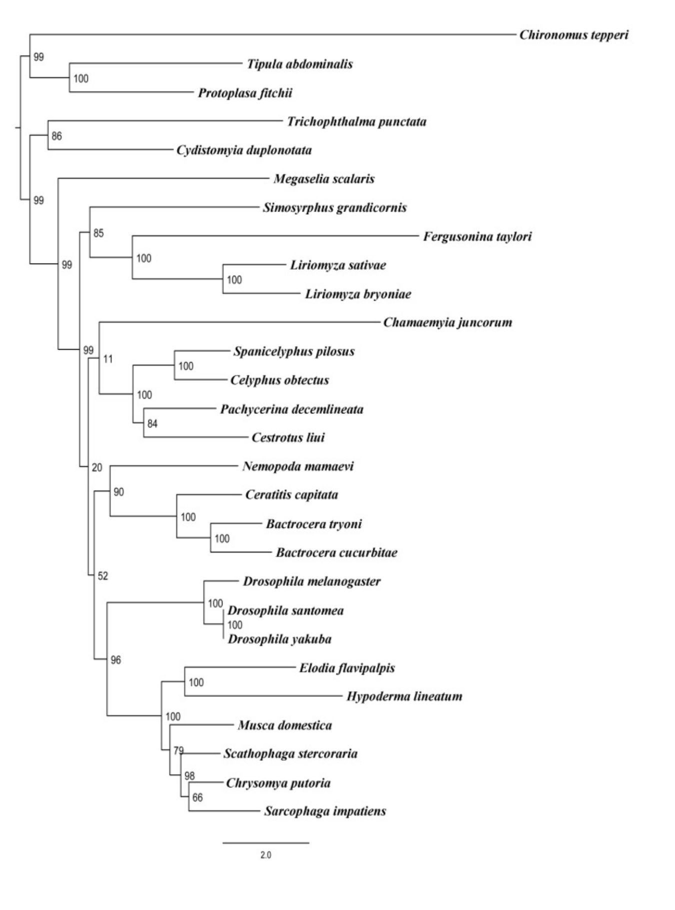

P123R_ML

Figure S1. Sequence aliments of three intergenic sequences among five lauxanioidean flies. A, the intergenic sequences between *ND1* and *tRNA*$^{Ser(UCN)}$, reversed sequences; B, the intergenic sequences between *tRNA*^{Glu} and *tRNA*^{Phe}, forward sequences; C, the intergenic sequences between *tRNA*^{His} and *ND5*, reversed sequences.

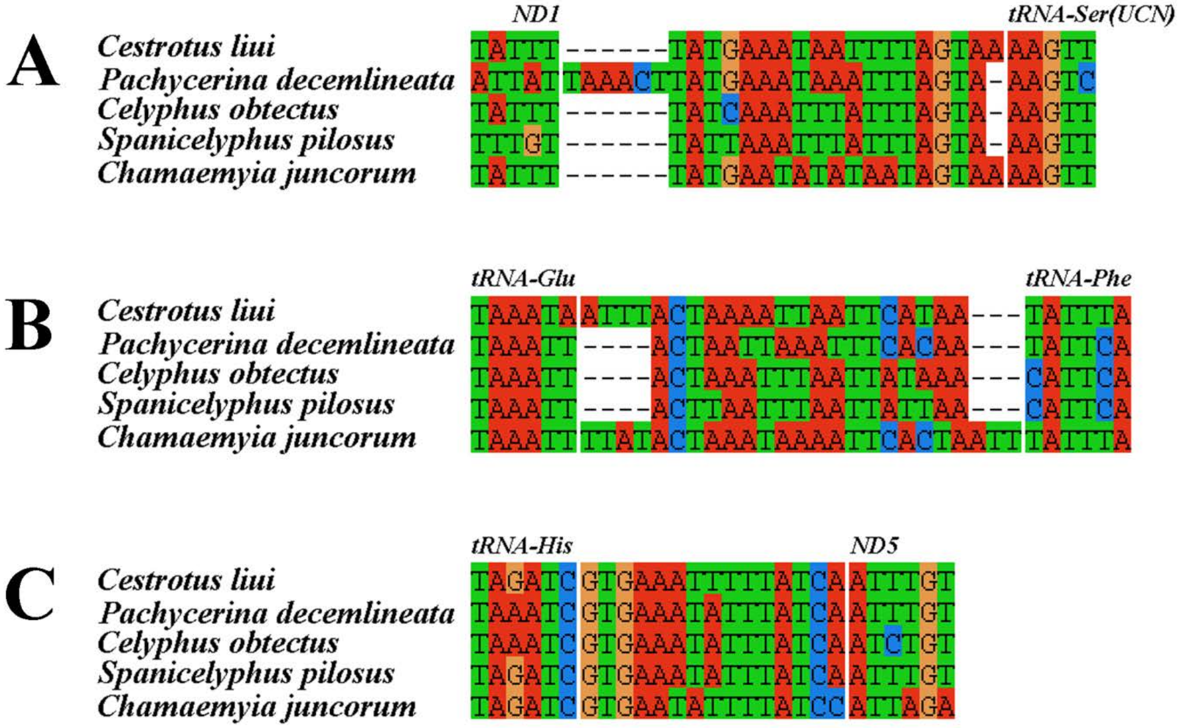

Figure S2. Putative secondary structures of tRNAs found in cyclorrhaphan mt genomes. Red filled circle, nucleotide conserved in cyclorrhaphan mt genomes; Green filled circle, nucleotide conserved in lauxanioidean mt genomes; hollowed circle, nucleotide not conserved. The cyclorrhaphan substitution pattern for each tRNA was modeled using as reference the structure determined for *Cestrotus liui*.

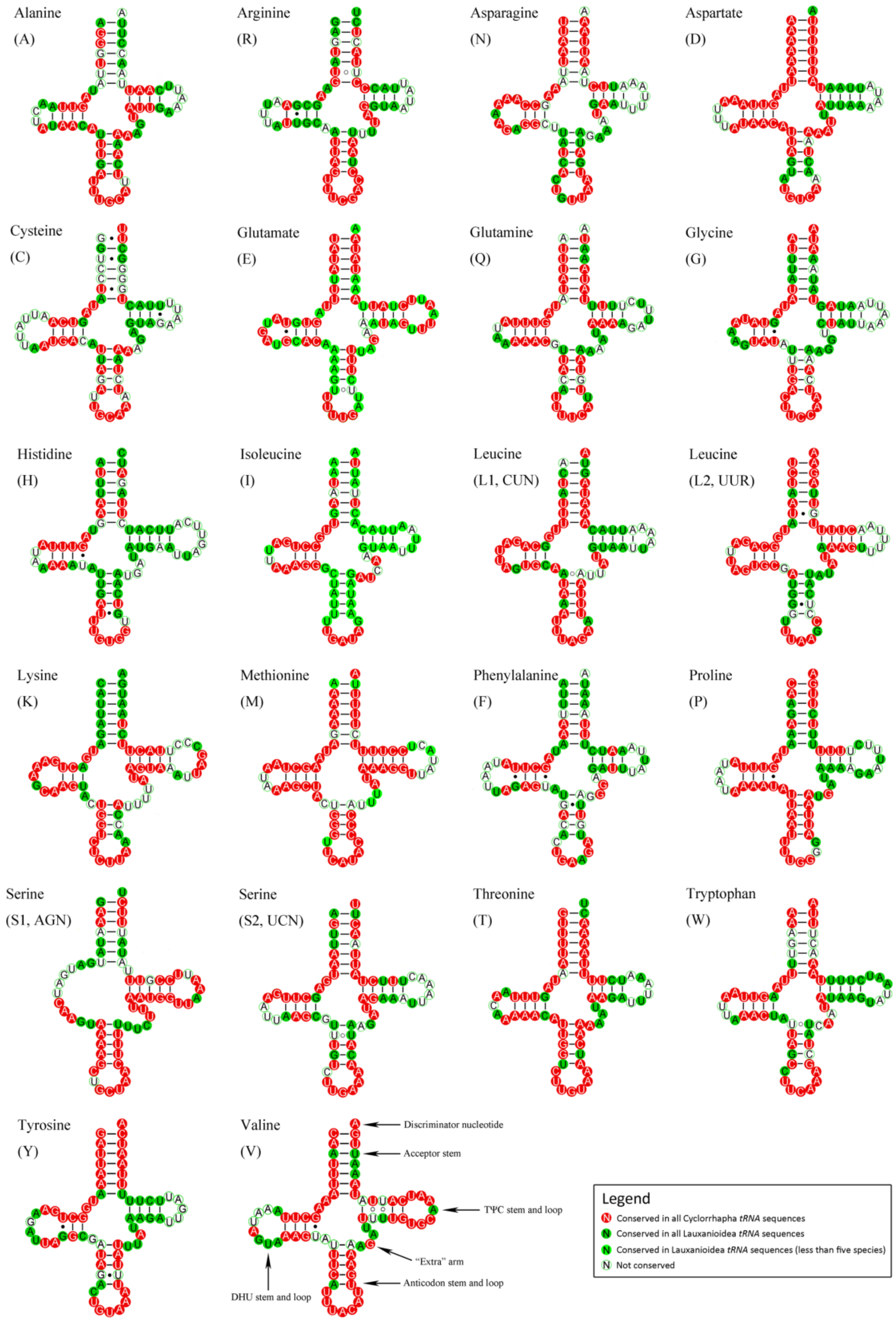

Figure S3. Conservation and A+T contents of tRNAs in cyclorrhaphan mt genomes. The percentage of identical nucleotides for each tRNA family was inferred from a multiple alignment produced with MEGA 5.0 (Tamura et al., 2011) and refined manually, taking into account the secondary structure.

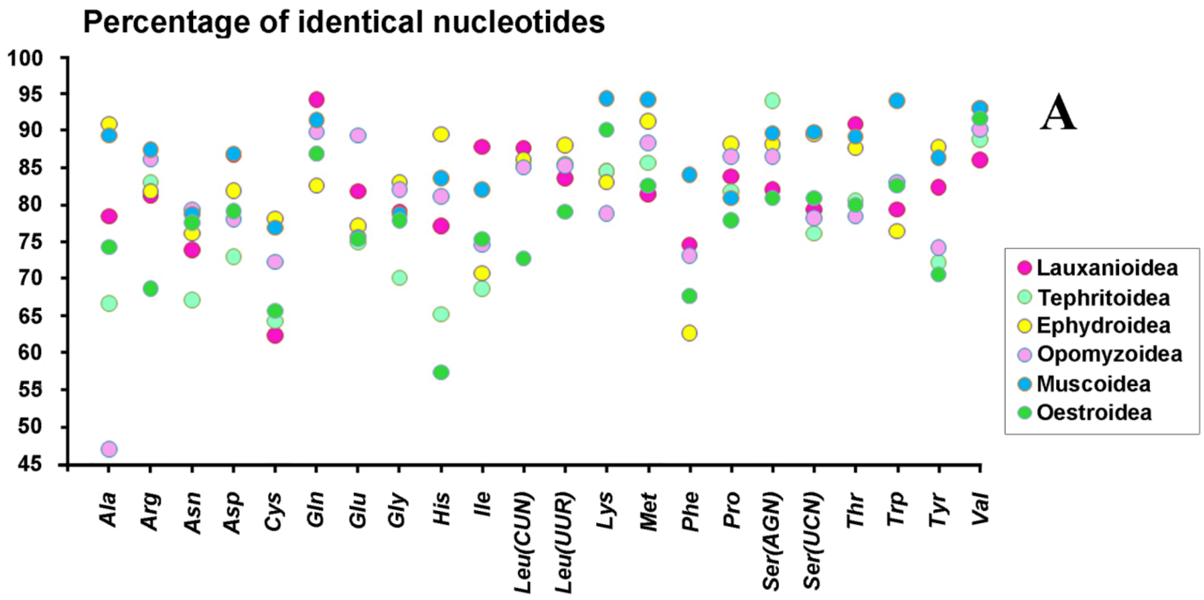

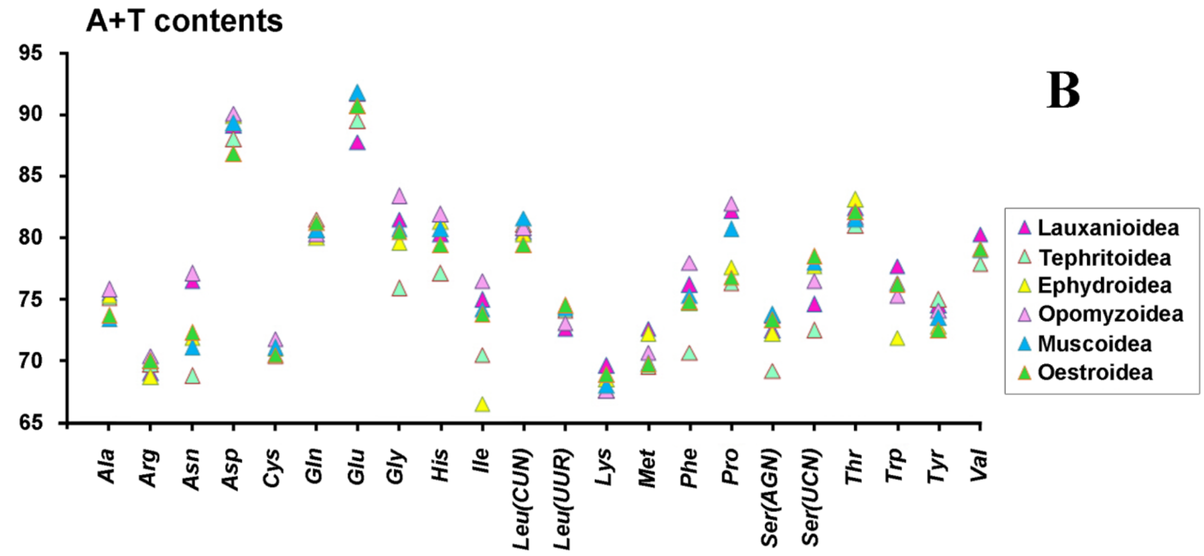

Figure S4. Predicted secondary structure of the *lrRNA* gene in *Cestrotus liui*. Red filled circle, nucleotide conserved in cyclorrhaphan mt genomes; Green filled circle, nucleotide conserved in lauxanioidean mt genomes; hollowed circle, nucleotide not conserved.

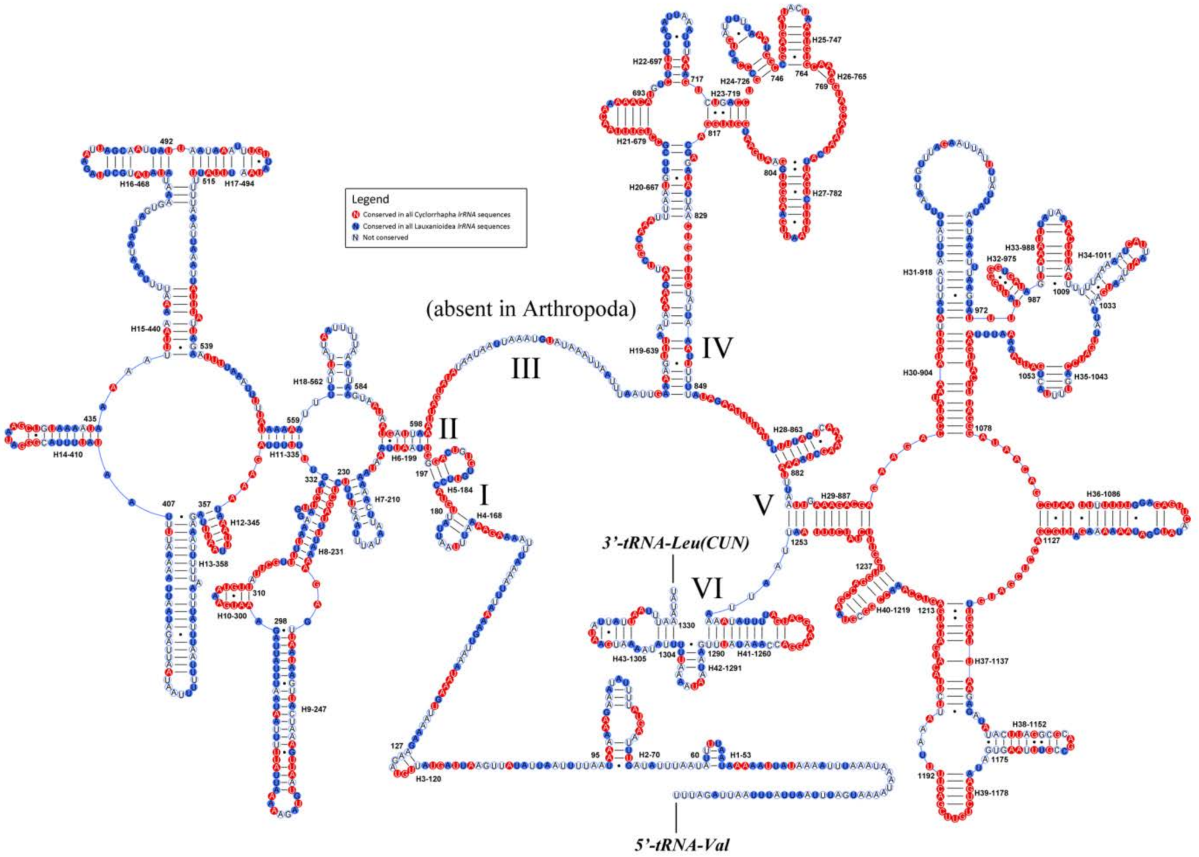

Figure S5. Predicted secondary structure of the *srRNA* gene in *Cestrotus liui*. Red filled circle, nucleotide conserved in cyclorrhaphan mt genomes; Green filled circle, nucleotide conserved in lauxanioidean mt genomes; hollowed circle, nucleotide not conserved.

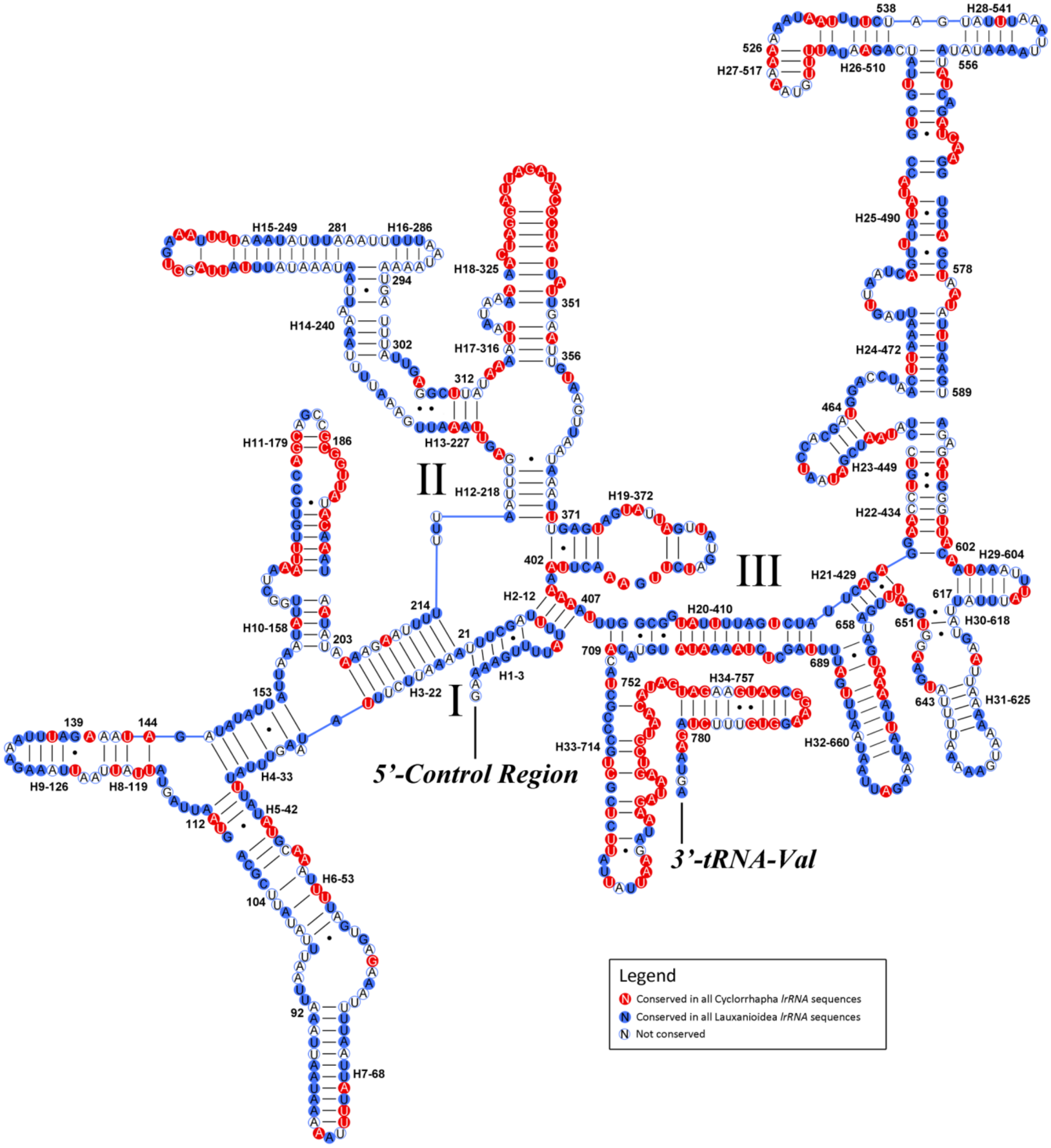

Figure S6. Nucleotides conservation and A+T contents of rRNAs in cyclorrhaphan mt genomes. Blue circle, percent of identical nucleotides; Red triangle, A+T contents.

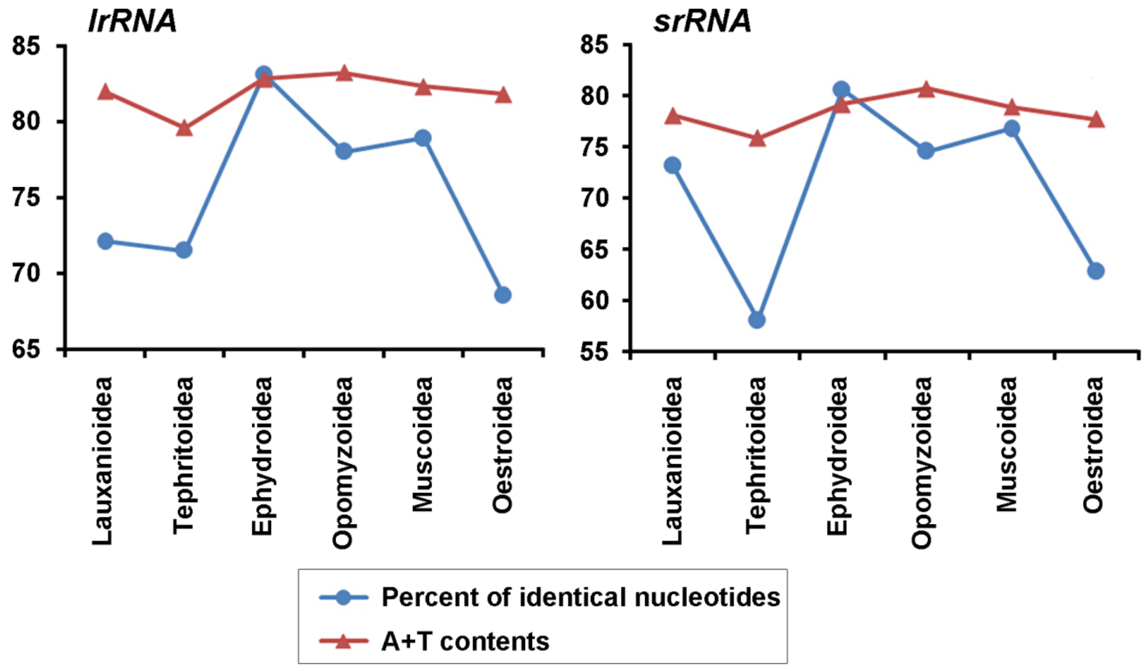

Supplement: Supplementary file 1 [file ijms-18-00773-s001.pdf]
